# Application of Genotyping-by-Sequencing for Analysis of Breeding Signatures in Russian Spring Barley Collection

**DOI:** 10.3390/ijms27209254

**Published:** 2026-10-18

**Authors:** Svetlana D. Afonnikova, Artem Yu. Pronozin, Antonina A. Kiseleva, Vjacheslav V. Piskarev, Tatyana V. Kukoeva, Andrey Ya. Sotnik, Gennady V. Vasiliev, Dmitry A. Afonnikov, Elena A. Salina

**Affiliations:** 1 The Federal Research Center Institute of Cytology and Genetics, Siberian Branch of the Russian Academy of Sciences, Novosibirsk 630090, Novosibirsk Region, Russia; 2 Siberian Research Institute of Plant Cultivation and Breeding—Branch of the Institute of Cytology and Genetics, Siberian Branch of the Russian Academy of Sciences, Krasnoobsk 630501, Novosibirsk Region, Russia

**Keywords:** barley collections, *Hordeum vulgare*, genotyping by sequencing (GBS), traces of breeding, domesticated barley diversity

## Abstract

The high adaptability of barley (*Hordeum vulgare* L.) facilitated its cultivation across almost the whole Russian territory and various extreme environments. However, the genetic diversity and population structure of historical Russian barley collections remain poorly characterized at the genomic level. This study provides the first comprehensive genomic description of the barley panel maintained in the GeneAgro seed bank. Using genotyping by sequencing (GBS), we obtained high-quality genotyping data of 158 Russian and 21 foreign accessions. We evaluated genome-wide diversity and population structure, revealing a clear genetic stratification into two-rowed and six-rowed barley. Russian accessions complemented the diversity of the more than 19,000 domesticated barley accessions from the Leibniz Institute of Plant Genetics and Crop Plant Research (IPK), expanding the representation of germplasm developed specifically for Russian environments and economic demands. To identify genomic regions that may have been shaped by breeding, we scanned the native GeneAgro barley accessions for selective footprints. We detected multiple such regions across the genome. Some were shared by two-rowed and six-rowed barley, while others were specific to one row type, which may reflect differences in their breeding histories. These regions contain candidate genes previously associated with spike architecture, plant morphology, flowering time, fertility, grain color, photosynthesis, seed dormancy and resistance to fungal pathogens. These candidates provide hypotheses for future allele-level and phenotypic studies.

## 1. Introduction

Barley (*Hordeum vulgare* L.) was domesticated from the Fertile Crescent over 11,000 years ago, and nowadays, it is one of the key cereal crops that is produced worldwide. Barley domestication was a long-term process of accumulating traits suitable for cultivation, such as naked caryopsis, six-rowed spike and non-brittle rachis [1,2]. Archaeological and genomic evidence strongly suggest a complex polycentric domestication history with recurrent introgression with wild barley [3,4,5]. Further expansion to Europe and West Asia revealed more territories with various climatic conditions that were the driving forces behind cultivation and divergence of barley varieties adaptable to specific environments [6,7]. Since the emergence of the six-rowed spike around 8000 years ago, barley cultivation acquired row type-specific directions, which led to extensive genomic differences between them [8,9,10].

The development of breeding techniques in the early twentieth century drastically accelerated the selection process of improved varieties with stable desirable traits [11]. This includes modern breeding programs that account for end-use properties of two- and six-rowed barley. For example, two-rowed barley is often preferred for malting due to its larger and more uniform grains and lower protein content; meanwhile, six-rowed barley is usually higher yielding and favored for animal feed because of its greater grain number per spike and higher protein content [12,13,14]. The past domestication events and breeding pressure applied to a particular group of varieties are reflected in the genome. Tracing these signals allows us to decipher unique genetic variants and novel genes that affect key agronomic traits and can be used as a source of genetic diversity essential for crop improvement [15,16,17,18].

The genetic characterization of one of the major barley gene banks hosted at IPK Gatersleben was described by Milner et al. using genotyping by sequencing (GBS) [19]. This approach provided a great power to capture genetic diversity among barley gene pools derived from around the globe and to detect known and novel loci associated with agronomic and adaptive traits. Nevertheless, this dataset still needs to be complemented by more focused studies of regional germplasms. In particular, cultivars and landraces from Russia remain underrepresented.

In this study, we aimed to describe the genetic diversity of a panel of domesticated spring barley hosted by the GeneAgro seed bank of ICG SB RAS underrepresented in the IPK gene bank. The panel included two- and six-rowed barley originated predominantly from Russian breeding centers. To trace the history of breeding efforts applied to GeneAgro Russian accessions, we investigated the genomic signatures of the selection process to find candidate genes targeted by breeders.

## 2. Results

### 2.1. Genome-Wide SNPs Discovery

Sequencing of 218 GBS libraries produced a total of 554.1 million reads, with a median of 2.55 million reads per library (Supplementary Figure S1A). Overall, 95.0% of reads mapped to the Morex V2 reference genome. The median mapping rate per library was 95.9% (range 64.6–99.4%), and 201 libraries (92.2%) had mapping rates of at least 90% (Supplementary Figure S1C). Consistent with the complexity reduction in GBS, reads covered a median of 72.6 Mb per library, about 1.7% of the genome. The median mean depth at covered positions was 2.52× (range 1.07–4.90×; Supplementary Figure S1B).

Filtering based on missing rate for individuals and SNPs resulted in total of 179 accessions and 18,687 biallelic SNPs. Observed heterozygosity across the 179 genotypes was low, as expected for a predominantly self-pollinating species: the median proportion of heterozygous calls was 0.021, and most genotypes fell between 0.015 and 0.035 (Supplementary Figure S1D). Among these 18,687 SNPs, 14,411 variants had a minor allele frequency (MAF) > 0.01, and 9250 variants had MAF > 0.05. All chromosomes showed a similar uneven distribution pattern, with less SNP concentration in the middle and more density at the terminal regions (Figure 1). For example, we observed a massive gap approximately from position 150 Mb up to 375 Mb on chromosome 4H and a large gap between 100 Mb and 275 Mb on chromosome 1H. While SNPs are present throughout the gene-rich regions, the density per bin is generally low to moderate, rarely reaching the high values of the scale.

### 2.2. Population Structure of GeneAgro Barley Panel

For population structure analysis of the GeneAgro panel, 179 accessions that remained after missingness filtration and SNPs with MAF > 0.05 were subjected to linkage disequilibrium (LD) pruning that resulted in a final set of 3181 SNPs.

Analysis showed that the first five principal components explained 67% of the total dispersion (PC1–PC5: 29%, 14%, 9%, 8%, and 7%, respectively, Figure 2B). The first principal component separated the samples into two clusters corresponding to two-rowed and six-rowed types of barley spike architecture (Figure 2A). However, we observed a few accessions cluster closer to the other row type (Supplementary Figure S2). For example, the two-rowed varieties #37 “Nepolegayushy”, #59 “Krasnokutsky”, #122 “Altan-Bulag”, #113 “Bagan”, and #90 “Ranny 1” were grouped with six-rowed genotypes, while the six-rowed varieties #120 “Dobry”, #152 “Zevs”, and #170 “Vakula” were positioned in a cluster of two-rowed samples. The phylogenetic tree replicated the clusterization of accessions by row type (Supplementary Figure S3).

Other components did not form distinct clusters, although certain patterns could be identified (Supplementary Figure S4). Along the second component, samples with Caucasus origin in Azerbaijan and Armenia were positioned at one end (57 “Pervenets”, 46 “Medicum 1”, and 34 “Agarpa”), while a large part of samples of Russian and European breeding centers, India, and the USA were concentrated at the opposite end.

The Cross-Validation error plot (Supplementary Figure S5) showed a clear elbow at K = 3 with a stable and reproducible value of 0.76. Then, on interval K = 4–9, the curve flattens and barely moves around 0.75–0.73, with weaker convergence. At higher K (>10), the curve goes up, indicating overfitting. We chose K = 3 as the optimal number of ancestral clusters because it aligned with the known population structure of the panel and demonstrated the lowest CV error with the smallest standard deviation between runs. At K = 3, the main genetic groups corresponded to accession geographic origin.

At K = 2, the inferred ancestral populations largely corresponded to row type: two-rowed accessions were predominantly assigned to cluster K1, whereas six-rowed accessions clustered primarily as K2 (Figure 2C). Consistent with the PCA, each row-type group contained a subset of individuals showing predominant ancestry from the opposite cluster. Specifically, some six-rowed accessions exhibited > 50% ancestry assignment to K1, which is prevalent in the two-rowed group. These accessions also grouped near the two-rowed cluster in the PCA scatter plot (Figure 2A). Conversely, four two-rowed accessions showed predominant K2 ancestry (K2 is prevalent in the six-rowed group), aligning with their proximity to the six-rowed cluster in the PCA.

A model with K = 3 genetic clusters resolved finer substructure for two-rowed barley, corresponding to geographic selection centers (Figure 2D,E; Supplementary Figure S6A,B with labeled individuals). Accessions from Armenia and Azerbaijan formed a distinct cluster assigned to genetic cluster K2. Ancestral cluster K3 was the main component in European two-rowed accessions (Figure 2D,E, Supplementary Figure S6A,B). The majority of six-rowed accessions remained assigned to cluster K1, indicating no further stratification at the K = 3 model. Accessions from Russia comprised six-rowed genotypes (K1) and two-rowed genotypes with mixed ancestry with both the Caucasus (K2) and European (K3) gene pools. 

### 2.3. GeneAgro Panel Projection onto the IPK Worldwide Barley Collection

The global collection of domesticated barley included 19,778 samples and 775,283 SNPs [20]. Merging with genotypes of the GeneAgro panel and filtering by the number of missing data points (< 20%) and minor allele frequency (MAF > 0.05) retained 18,342 samples and 7980 SNPs. The IPK biobank possessed 218 Russian samples comprising mostly six-rowed barley and winter growth varieties. In turn, the GeneAgro panel included only spring barley with prevalent two-rowed spike architecture, filling out the Russian barley diversity.

In the context of global barley diversity, domesticated Russian accessions from GeneAgro clustered together with most Russian germplasm from IPK (Figure 3A). At the same time, within the subgroup of all Russian samples, specific patterns of clustering were observed (Figure 3B). Along the first component, multiple six-rowed winter barley samples from the global collection formed their own separate cluster. Along the second component, no clear separation between groups was observed; however, samples created two dense clusters on opposite sides of the component. Notably, these clusters were shaped according to row type, where the GeneAgro panel accounted for most of the two-row representation.

Thus, the PCA results have shown that domesticated Russian barley from GeneAgro complements genetic diversity both in the global barley collection and in the Russian varieties available in the IPK biobank.

### 2.4. Breeding Signatures of Russian GeneAgro Barley

To identify regions targeted by historical and recent breeding, we integrated in-population (nSL and Tajima’s D) and cross-population (Fst, XP-nSL) statistics across two- and six-rowed groups of the GeneAgro Russian accessions. The division into two- and six-rowed groups was performed based on the first PCA component (Figure 2A). To obtain more powerful results, we excluded samples that clustered closer to the opposite row type group. The number of SNPs and bins that were processed by the methods and then retained after filtration are shown in Supplementary Table S1. Detailed information about candidate regions is presented in Supplementary Table S2 (regions obtained after the SweepCluster process described in Section 4.7, Search of candidate genes targeted by breeding, in Materials and Methods). The final selection signals-scan dataset comprised 143 accessions: 115 two-rowed and 28 six-rowed. All retained accessions originated from Russia. Both groups were dominated by advanced cultivars, split roughly evenly between historic (pre-1990 admission) and modern (post-1990) releases.

Using the Tajima’s D test, we discovered 25 and 20 highly significant regions with extreme values for two- and six-rowed accessions, respectively (Figure 4A,B). The majority of the signals possessed positive values. In two-rowed barley, they were located on all chromosomes except 1H, while the six-rowed group carried positive Tajima’s D on all chromosomes except 6H and 7H. Positive Tajima’s D value usually means an excess of medium-frequency alleles due to balancing selection favoring both alleles or due to bottleneck in the population. To distinguish these explanations, further analysis of allele-level data would be required. Each group also displayed regions with negative Tajima’s D values: in two-rowed barley, these were located on chromosomes 1H, 2H, 3H, 6H, and 7H, while in six-rowed barley, they spanned chromosomes 1H through 5H, inclusive. Negative signals indicate sites with reduced allelic diversity.

Both groups shared breeding signatures on chromosome 1H near 514 Mb, on chromosome 2H around 600 Mb, a common negative signal on 3H (553 Mb), as well as two regions on 5H around 590 Mb and at a locus spanning from 517 to 565 Mb. In the six-rowed group, the largest number of regions deviating from neutral evolution was found on chromosomes 1H and 5H. Meanwhile, in two-rowed barley, chromosomes 3H, 6H, and 7H were the most enriched with significant selection signatures.

Significant nSL results of each group were scattered across all seven chromosomes and clustered into 34 and 24 regions for two- and six-rowed barley, respectively (Figure 5A,B). Significant positive nSL values indicate sweeps favoring the alternative allele (labeled as “1”), while a negative value means possible selection to maintain the reference state (labeled as “0”).

The general distribution of recent selective sweeps across chromosomes showed row-type-specific patterns, with eight regions (four on 5H and one each on 1H, 2H, 6H and 7H) shared between groups. Two-rowed barley showed strong signals on chromosomes 1H, 2H, 5H and 7H, while six-rowed genotypes carried extended haplotype regions on chromosomes 1H, 3H and 5H.

The majority of signal-dense regions included both negative and positive sweeps, and their size, in general, equaled to 6 Mb, while the maximum length reached approximately 28 Mb on chromosome 5H of six-rowed barley.

Genomic regions with allele frequencies differentiating between two- and six-rowed barley were revealed by fixation index Fst (Figure 6A). Unusually high Fst values in a particular region indicate high genetic divergence between groups, possibly due to different breeding directions. Cross-group haplotype scan XP-nSL was applied to explore local adaptation targets, where recent positive selection acts in one population but not in another (Figure 6B). In this study, six-rowed barley was used as the reference population and two-rowed as the target. Thus, positive values indicate a recent selection in two-rowed genotypes, whereas negative values infer selection sweep in six-rowed ones.

The genome average weighted Fst value equaled 0.213 with maximum up to 0.817. The top 5% strongest signals were located on chromosomes 2H, 3H, 5H, 6H and 7H. The XP-nSL analysis highlighted positive extended haplotype regions on chromosomes 4H, 5H and 6H. Other prominent peaks were negative and located on chromosomes 2H, 5H and 7H.

Three regions displayed both recent sweeps and elevated site frequency divergence (chromosomes 2H, 5H and 7H). Among these, weighted Fst value ranged from 0.7 to 0.82, corresponding to strong population differentiation.

### 2.5. Candidate Genes Targeted by Russian Breeding

We conducted the gene search based on significant regions identified by two or more selection footprint analyses (Supplementary Table S2). A list of genes cloned by the forward genetics approach provided by Hansson et al. [21,22] was used as a source of functionally validated genes/loci. We considered a gene as a candidate if its alignment on Morex v2 coordinates lay within the region with 5 Mb interval from boundaries. In total, we identified 32 well-known candidate genes we propose as hypothetical breeding targets (Table 1). They participate in a wide range of plant physiological and morphological traits such as row type, spike architecture, grain color, fertility and tolerance to biotic stress.

## 3. Discussion

### 3.1. Russian Barley Expands the Genetic Diversity of the IPK Worldwide Barley Collection

Although barley germplasm is conserved in many major gene banks, genotypic data is publicly available for only a small fraction of these accessions [23]. Among the GBS studies in barley summarized by Rajendran et al. [23], most used biparental mapping populations or introgression lines. The Leibniz Institute of Plant Genetics and Crop Plant Research (IPK) collection, therefore, remains the only large-scale and openly available GBS resource representing a major barley gene bank. Smaller publicly available panels, such as Canadian six-rowed lines [24], could be integrated in future work. Extending the analysis to other gene bank collections will be become possible as their genotyping data is released.

The GeneAgro spring barley panel comprising foreign cultivars and Russian modern cultivars and landraces from various breeding centers presents a valuable resource of genetic diversity. For the first time, the panel was genotyped using the genotyping-by-sequencing approach. This method allows discovery of novel single-nucleotide polymorphisms (SNPs) across the whole genome. Typically, the overall SNP distribution is uneven: sparse in the middle and denser at the gene-rich meaningful ends due to DNA cutting enzyme sensitivity to methylation [25,26]. 

We were able to explore the genetic diversity of the GeneAgro panel on a scale of extensive collection of more than 19,000 domesticated barley accessions from IPK [19]. Out of 218 IPK accessions of Russian origin, only 62 genotypes displayed a spring growth habit, including 33 two-rowed types. Principal component analysis showed that the GeneAgro accessions overlapped with and complemented the distribution of the Russian IPK accessions.

Moreover, among the Russian samples from both panels, we observed a tendency to cluster by row type: the IPK group consisted predominantly of six-rowed genotypes, while the GeneAgro panel formed a denser cluster of spring two-rowed genotypes. This finding indicates that the GeneAgro panel provides a valuable genetic resource to expand the currently limited diversity of two-rowed spring barley of Russian origin. 

### 3.2. Row Type Is the Primary Driver of Genomic Divergence in Russian Barley

Because two-rowed and six-rowed barley is typically bred as two independent populations with distinct end-use objectives, the genome exhibits massive differentiation between them, especially in modern cultivars [10,27].

We observed this differentiation on a population scale. Cultivars were a dominant group in the GeneAgro panel and shaped the genetic population structure based on row type. In this work, a few landraces that passed the quality control followed the row type clusterization, although typically, landraces maintain heterozygosity and standing variation compared with cultivars [28,29]. We suppose that landraces lacked enough power to form a distinct cluster, probably due to the small number left.

Two-rowed and six-rowed barley face different breeding pressures due to different end-use demands [30]. Two-rowed barley breeding programs target malting-related traits such as lower protein content, plumper and more uniform grains and grain composition [31]. Six-rowed barley also can be used in the malting industry; however, more often, practices employing this row type include animal feed and food production [32]. The long history of these different breeding scenarios left genomic changes (such as allele and haplotype frequencies) that affect genes determining traits of interest [10]. Nevertheless, these row types share target genes. Usually, they are key factors that influence adaptation and ability to expand habitat territory, plant growth and yield [10,15,33]. These traits include vernalization response, photoperiod sensitivity, disease resistance and optimal plant architecture. In the current study, breeding signatures analysis combined multiple methods, increasing the power to discover highly reliable genomic regions affected by breeding [34]. Inside them, we found multiple candidate genes responsible for the traits above, i.e., grain quality and reproduction.

We note that the two groups differ substantially in size (115 vs. 28 accessions), which limits direct comparison between them. The six-rowed group, in particular, is small and may provide limited statistical power of analysis. That is why, for the candidate loci search, we considered regions that were detected by multiple methods. Additional requirements (such as number of statistically significant SNPs per region) further increased confidence in these regions.

Both groups are overwhelmingly composed of advanced cultivars rather than landraces, which likely explains the recovery of well-characterized domestication and breeding-target loci (spike row type, flowering time) as the dominant breeding signatures. This pattern is consistent with a cultivar-enriched sampling structure. Nonetheless, we view the recovery of the well-established breeding loci as a meaningful validation step rather than a limitation of the analysis. Because this panel has never previously been genotyped, correctly detecting the expected signals at row-type, spike-architecture, and flowering-time loci confirms that our data and analysis pipeline work as intended. This is an important first check before using this dataset to study less-characterized traits in future work. Thus, the present analysis both validates the resource and establishes a genotyped baseline for the panel that did not previously exist, providing a foundation for subsequent, more targeted studies.

#### 3.2.1. Spike Architecture 

As expected, we found multiple genes involved in spike morphology. Tajima’s D test for six-rowed barley and both in-group nSLs identified significant signals near the *Int-m* gene (HORVU.MOREX.r3.5HG0525620) encoding APETALA2-like transcription factor HvAP2L-H5, which promotes inflorescence meristem activity [35]. In barley, this factor inhibits floral differentiation genes, resulting in indeterminant inflorescence meristem and single-flowered spikelets. Mutations in the *HvAP2L-H5 (Int-m)* gene can lead to a phenotype intermediate between two- and six-rowed types [35].

Chromosome 5H carried multiple breeding signatures containing other spike architecture genes *HvCOM1* (HORVU.MOREX.r3.5HG0479720), *HvDEP1* (HORVU.MOREX.r3.5HG0480200) and *HvRAW1* (HORVU.MOREX.r3.5HG0502720). In barley and wheat, *HvCOM1* regulates the spikelet meristem morphology, ensuring unbranched spike architecture [36]. Loss of COM1 causes spikelet meristems to revert into inflorescence meristems, resulting in a highly branched phenotype. Cytokinin riboside 5′-monophosphate phosphoribohydrolase encoded by the *HvRAW1* gene affects spike morphology by increasing cytokinin content in the awn primordia, initiating the growth of sharp barbs [19,37]. Complex selection history of awn roughness (shown by nSL, XP-nSL and Tajima’s D signals) may correspond to trait ambiguous directions during domestication and breeding. Rough awns benefit barley by providing defense against insect herbivores, overheating and other environmental stresses in the wild [37,38]. On the other hand, in agriculture, it challenges harvesting and grain storage and may cause health issues for livestock animals [39]. *HvDEP1* acts as a positive regulator of stem elongation and grain size [40]. Mutants display semi-dwarfism, smaller and rounder grains, shorter awns and reduced thousand-grain weight [40]. Although this phenotype protects from lodging, the overall grain yield is highly environment-dependent, requiring more precise breeding with consideration of the narrow range of cultivation territory.

On chromosome 1H, we detected a Tajima’s D signal near *THIRD OUTER GLUME 1* (*Trd1*, HORVU.MOREX.r3.1HG0091840) for both row types. *Trd1* encodes a GATA-type transcription factor, an ortholog of rice *NECK LEAF1* and maize *TASSEL SHEATH1* [41]. Normally, the gene participates in bract outgrowth suppression at the base of the spike during the floral phase. Apparently, the suppression plays a role in redirecting resources toward axillary meristems of the inflorescence and spikelets [42,43]. In *trd1* mutants, this suppression fails, and an extra glume-like bract develops at the spike base; however, floral development at the collar is not necessarily suppressed [41].

Cross-group haplotype-sharing analysis XP-nSL detected ongoing sweep near the *HvCMF4* gene HORVU.MOREX.r3.4HG0411680 in two-rowed barley. Meanwhile, the significant positive Tajima’s D signal in six-rowed barley suggests a retained genetic diversity at this locus. HvCMF4 is a grass-specific (Pooideae) protein from CCT MOTIF FAMILY 4, and it is involved in light signaling and circadian clock regulation, which control chloroplast biogenesis and vascular morphogenesis [44]. The loss of *HvCMF4* function in the *tst2.b* mutant causes impaired plastid development and reduction in vein size, leading to a decreased xylem transport capacity at the spike apex [45]. Thus, apical spikelets deprived of adequate sustention die in a top-down manner, reducing the grain number per spike.

We were able to detect the main row type determinants [9,46], although obtained by a single statistic. The *vrs4* (HORVU.MOREX.r3.3HG0233930) was located closely between two Tajima’s D regions for two-rowed barley (roughly at 6 Mb distance from the second signal), while *vrs1* (HORVU.MOREX.r3.2HG0184740) was discovered to be significant by haplotype in-group scan inside two-rowed accessions. As one of the possible explanations, we suppose that these loci lacked enough SNP density to pass strict filtration requirements on SNP number per region or failed to reach the rather high threshold value obtained by other methods. For instance, the Fst value near *vrs1* coordinates equaled 0.42, which is higher than the genome average, however, is unsatisfactory to the top 5% threshold (not provided). Meanwhile, nSL on six-rowed barley detected only two SNPs with normalized nSL = –2 near *vrs4*, which is not enough to declare the region significant. Although strict filtration allows obtaining the most reliable regions, it may miss some true biological results due to technical interference. Another explanation lies in the panel itself. The power of the applied methods was limited due to the unbalanced dataset with predominant two-rowed group. An increase in sample size would also improve the precision of analyses.

#### 3.2.2. Plant Morphology

Two shoot morphology genes were located on chromosome 7H. In two-rowed barley, we traced more profound breeding history in two-rowed barley near the *HvDL* (HORVU.MOREX.r3.7HG0736060) and *HvDIM* (HORVU.MOREX.r3.7HG0749940) genes. Multiple Tajima’s D, specifically to two-rowed accessions, may indicate an earlier selective process at the locus for this group. For six-rowed barley, our results demonstrate that breeding pressure was applied on the locus only recently because of the negative XP-nSL and two in-group nSL signals. *HvDL* encodes the transcription factor of the ALOG family, and HvDIM protein participates in brassinosteroid synthesis and signaling pathway [47,48]. Mutant forms of both genes demonstrate semi-dwarf stature and changes in leaf structure and shape.

On chromosome 2H, we identified the *ELIGULUM-A* (*ELI-A*) gene that acts as a critical boundary specifier during leaf development [49]. *ELI-A* encodes a previously unannotated protein containing a conserved RNase H-like domain, and while homologs exist across land plants, their specific developmental functions remain largely uncharacterized even in Arabidopsis. Loss or alteration of *ELI-A* function results in leaf boundary and cell wall defects as well as overall plant height (semi-dwarf stature) and compact spikes with tightly packed spikelets [49]. 

Negative Tajima’s D values suggested that the low number of tillers-1 (Lnt1, HORVU.MOREX.r3.3HG0302040, chromosome 3H, was critical itself or closely linked to a critical unidentified gene for both row types. *Lnt1* encodes a BELL-like homeodomain transcription factor, homologous to Arabidopsis BELLRINGER, and is required for normal growth of axillary buds into tillers [50,51]. Recessive *lnt1* mutants do not develop secondary tillers and produce only one to four tillers by maturity [51].

In general, plant morphology genes serve as targets to engineer compact, upright canopies for high-density planting, but their pleiotropic effects on fertility, culm strength, and grain size require precise editing or allele selection rather than simple loss of function [52].

#### 3.2.3. Growth Habit and Reproduction

Growth habit and reproduction are key breeding targets because they directly determine a crop’s yield potential and adaptation to specific environments [53,54,55]. The time of reproduction processes strongly depends on a network of vernalization (*Vrn*) and photoperiod (*Ppd*) genes. In the present study, the major long-day response factor Ppd-H1 (*HvPRR37*, HORVU.MOREX.r3.2HG0107710) was detected by nSL for two-rowed barley and by Tajima’s D for six-rowed. In southern latitudes, the dominant allele maintains the response to day length increase and ensures early flowering, allowing the plant to avoid drought and heat [56]. The recessive *ppd-H1* allele promotes late flowering beneficial in northern high-latitude environments with very long days [57]. Delayed flowering prevents too early maturing, allowing biomass accumulation and higher yields in cooler and wetter summers [58]. The allelic state at *Ppd-H1* and selection directions cannot be interpreted apart from the vernalization and geographic origin [58]. For instance, among Russian cultivars sharing identical *Ppd* alleles, carriers of *Vrn-H1 vrn-H2 Vrn-H3* headed significantly earlier than other *Vrn* combinations [59]. In addition, the recessive *ppd-H1* allele was prevalent in spring accessions from Russia and Belarus [59]. Thus, more detailed inspection of specific *Ppd* and *Vrn* alleles inside the GeneAgro panel would allow restoring their breeding history.

The GeneAgro all-spring barley panel carried extensive breeding signatures for both row types near the region on chromosome 5H carrying *HvPHYTOCHROME C* (*HvPHYC*, HORVU.MOREX.r3.5HG0511090) and *Vrn-H1* (HORVU.MOREX.r3.5HG0511210) genes. Furthermore, significant nSL signals together with multiple negative Tajima’s D for six-rowed barley indicate a recent sweep in the group. *HvPHYC* encodes a red/far-red light photoreceptor that transmits light signals to the plant’s internal circadian clock [5,60]. Mutation in a conserved region of the GAF domain in the *HvPHYC* gene accelerates flowering and heading dates under both long and short days [5,61]. *Vrn-H1* is considered a primary locus for cold-temperature adaptation and the transition from vegetative to reproductive growth in barley [62]. Spring and winter barley carry two distinct haplotypes, and deletions and rearrangements in the first intron lead to bypassing the vernalization requirement and spring growth habit [53]. The second vernalization gene, *Vrn-H3* (HORVU.MOREX.r3.7HG0653910), is the barley ortholog of Arabidopsis FLOWERING LOCUS T (FT) [63]. *HvFT* produces a transmissible signal that moves from leaves to the shoot apex to trigger flowering [64]. In barley, *HvFT* transcript levels are significantly higher in plants carrying the dominant, early-flowering *Vrn3* allele than the recessive, late-flowering allele [63]. Our results demonstrated a selective sweep of the locus in two-rowed GeneAgro accessions in accordance with negative Tajima’s D and extended haplotype homology analysis.

Another flowering time gene, EARLY MATURITY 8 (EAM8, HORVU.MOREX.r3.1HG0095050), is mapped to the long arm of barley chromosome 1H inside borders of genomic region detected by Tajima’s D for both row types. It encodes the barley ortholog of Arabidopsis ELF3 [65,66]. The gene comprises four exons, and its product is a large protein that carries no recognized structural domain [67,68]. It is intrinsically disordered, with N-terminal (N), middle (M) and C-terminal (C) regions that mediate interaction with various proteins [67,69,70]. It appears to function as an interaction hub rather than as an enzyme, acting within the evening complex to repress clock- and growth-associated transcription factors [71]. All characterized *eam8* alleles are recessive and disrupt the protein [65,66,67,68]. The consequences are consistent across alleles: mutants flower early, independent of day length, which leads to biomass and tiller number decrease.

Multiple significant signals revealed three genes involved in fertility: *HvLEAFY* (*HvLFY*, HORVU.MOREX.r3.2HG0194240), *HvMLH3* (HORVU.MOREX.r3.5HG0503940) and *Cly1* (HORVU.MOREX.r3.2HG0204770). We observed ongoing selection at these loci in both row types, particularly stronger in six-rowed barley (indicated by negative XP-nSL values). *HvLFY* encodes a transcription factor that regulates floral meristem identity [72]. Loss of the function allele in the multiovary 5 (*mov5.o*) mutant results in various floral disruptions, including formation of multiple carpels and ovaries and mosaic floral organs. Despite the majority of florets possessing abnormalities, a few occasionally develop an ovule and functional embryo sac. *HvMLH3*, in turn, functions on the cell level. This gene encodes a MutL-Homolog 3 protein, a crucial mismatch repair protein involved in the resolution of meiotic crossovers [73]. An impaired protein fails to form a functional endonuclease complex involved in Holliday junctions resolving, and consequently, causes loss of crossovers and chromosome mis-segregation [74,75]. The latter produces genetically unbalanced tetrads and, consequently, a semi-fertile phenotype [76]. The potential implication of the *MLH3* gene in rice was proposed by Mao et al. [74]. They showed that *OsMLH3* knockout lines produce fertile pollen but aborted embryo sacs, enabling their use as female-sterile restorer lines for mechanized hybrid seed production. To this day, barley *HvMLH3* knockouts alone appeared to show no beneficial effects on barley improvement. However, genes like *HvMLH3* are important as levers to understand and re-engineer recombination paired with other genes such as anti-crossover target *HvRECQL4* [76]. 

The cleistogamy 1 (*Cly1*, HORVU.MOREX.r3.2HG0204770) is located on chromosome 2H. *Cly1* was shown to encode a transcription factor related to the Arabidopsis *AP2* gene [77]. Active *Cly1* is associated with inhibition of lodicule development, leading to closed florets and cleistogamy [77,78]. Cleistogamous barley self-pollinates before the floret opens, reducing cross-pollination, which could be applied in breeding to force self-pollination.

#### 3.2.4. Pigmentation

Four candidate genes are involved in pigment biosynthesis, which, in turn, is associated with grain quality and yield. On chromosome 2H in neighboring robust selection regions, we detected *Ant2* (*HvbHLH1*, HORVU.MOREX.r3.2HG0188710) and *Ant17* (HORVU.MOREX.r3.2HG0201360) genes, chromosome 3H carried *Ant28* (HORVU.MOREX.r3.3HG0316260), while chromosome 5H contained signal near *Ant30* (HORVU.MOREX.r3.5HG0525800). Among these, genes *Ant2* and *Ant28* encoded transcriptional factors [79,80], and the other two carried information about structural enzymes of flavonoid biosynthesis [81,82].

Differentiation indices and haplotype extension analyses indicate that breeding efforts targeted both row types. The *Ant2* gene encodes basic helix-loop-helix (bHLH) transcription factor that regulates the expression of key genes involved in the flavonole pathway, producing purple and blue anthocyanins [83]. A premature stop codon upstream of the basic helix-loop-helix domain knocks out the gene’s function and leads to a non-pigmented white phenotype across plant tissues. Some mutations, however, may produce a more complex phenotype. Shoeva et al. screened 504 barley accessions and described six different *Ant2* alleles [80]. Among them, allele *Ant2.l* drives much stronger expression in grain tissues and provides purple coloration, while others are sufficient for pigmentation in vegetative tissues but fail to trigger pigmentation in the grain.

*Ant28* encodes R2R3 MYB transcription factor, homologous to the wheat *Tamyb10* gene, that regulates genes required for accumulation of proanthocyanidins in the developing seeds [79]. Notably, mutation in *Ant28* erases proanthocyanidin only in grain tissues, while vegetative ones maintain a wild-type proanthocyanidin content. *Ant17* and *Ant30* encode the flavonoid pathway enzymes flavanone-3-hydroxylase and chalcone isomerase, respectively [81,82]. Mutations in these two genes inhibit anthocyanin synthesis in the vegetative tissues of barley and proanthocyanidin synthesis in seed outer layers [82,84].

Notably, both *Ant17* and *Ant28* were found to be under ongoing selective pressure in two-rowed barley, supported by negative Tajima’s D and nSL. Two-rowed barley nSL analysis also demonstrated recent sweeps near *Ant30*. Meanwhile, combination of positive Tajima’s D and present nSL near *Ant2* and *Ant30* for the six-rowed group indicated recent breeding on multiple average-frequency variants. It should be noted that these genes were linked to other possible breeding targets, while *Ant28* and *Ant17* were isolated.

We suggest that because two-rowed barley is predominant in the brewing industry, breeding programs target genes involved in malting quality selecting on specific alleles [85]. Proanthocyanidins from the barley coat influence the brewing process by forming complexes with proteins that reduce beer’s shelf life [85]. Targeting *Ant28* in particular may solve the issue, but it poses a risk of reduced dormancy and consequent pre-harvest sprouting susceptibility. *Ant17* and *Ant30* participate in later proanthocyanidin synthesis stages, and their alteration may result in an effect that is more specific. Rather than complete knockout, a partial suppression of the proanthocyanidins pathway is more effective in controlling negative impacts on the plant’s vegetative tissues and yield-related traits [86]. Dissecting independent genetic mechanisms of agronomic traits would allow compensating for side effects.

#### 3.2.5. Stress Resistance

Resistance to biotic stress induced by fungal diseases is one of the main breeding targets in crops. The GeneAgro accessions carried multiple significant breeding signatures near genes involved in resistance to stem rust, leaf rust and powdery mildew. These diseases pose a threat to barley cultivation around the world, reducing grain quality and causing dramatic yield losses.

Stem rust is caused by the fungus *Puccinia graminis* f. sp. *tritici*, and for a long time, *Rpg1* was the only protection factor against the disease; the emergence of new, robust fungus races demanded a search for more genes [87]. As a result, studies found two linked genes, *Rpg4* (HORVU.MOREX.r3.5HG0526170) and *Rpg5* (HORVU.MOREX.r3.5HG0526140), encoding highly specialized, chimeric, and structurally unique immune receptors [88,89]. Our study revealed ongoing selection in regions harboring these two genes for both row types, indicating an extensive breeding targeting adaptation to the evolving pathogen.

Another *Puccinia* member, *Puccinia hordei* Otth, induces a leaf rust disease in barley leaf blades and sometimes on leaf sheaths [90]. Barley possesses multiple genetic mechanisms of resistance to the constantly evolving pathogen, where *Rph1* (HORVU.MOREX.r3.2HG0100980) and *Rph3* (HORVU.MOREX.r3.7HG0741050) genes, in particular, were the key breeding targets in GeneAgro Russian barley. *Rph1* encodes a coiled-coil nucleotide-binding leucine-rich repeat (CC-NLR) receptor protein that recognizes the fungus and induces a cascade of cellular processes leading to cell death and the pathogen’s starvation [91]. *Rph3* encodes a highly divergent transmembrane protein that is transcribed only in the presence of *P. hordei,* triggering localized cell death to stop the pathogen [92]. Interestingly, the *Rph3* gene is believed to have originated in wild barley and then transferred to domesticated germplasm [92].

In the GeneAgro panel, only two-rowed barley showed in-group breeding signals carrying the *Rph1* gene. Accounting for the positive value of Tajima’s D, we suggest that the two-rowed group maintained multiple variants that provide more flexible resistance to leaf rust. Near *Rph3,* we also observed significant positive Tajima’s D in two-rowed accessions. Although the low genetic diversity of the *Rph3* region in domesticated germplasm was expected [92], the presence of standing variation in Russian GeneAgro accessions sparks interest to further study the genetic diversity of the panel. Extended homozygosity haplotypes (negative XP-nSL) and recent sweep signal for the six-rowed group suggest that breeding programs of Russian six-rowed barley appear to have targeted the *Rph3* gene recently.

Finally, we detected an extensive breeding process targeting *RAR1* (HORVU.MOREX.r3.2HG0189710) and *ROR2* (HORVU.MOREX.r3.5HG0517040), genes involved in resistance against powdery mildew disease caused by the fungus *Blumeria graminis* f. sp. *hordei* [43]. *RAR1* encodes protein with a highly conserved zinc-binding domain structure. *Rar1* homologs were found in protozoa, metazoa, plants and fungi, except yeast [43]. Together with SGT1 and the molecular chaperone HSP90, RAR1 forms a protein complex that is required for the accumulation and function of many resistance (R) proteins that trigger race-specific plant immunity [93,94,95]. *ROR2* encodes a syntaxin shown to mediate penetration resistance of the plant cell wall [96]. In the gene net that regulates powdery mildew resistance, *ROR2* is required for proper function of multiple *Mlo* genes that offer durable, broad-spectrum mildew resistance [97]. Selective signals capturing these genes indicate an active breeding process to improve barley adaptation to this disease in both row types.

#### 3.2.6. Photosynthesis

We detected two photosynthesis genes: *Xantha-g* (*Xnt-g*, HORVU.MOREX.r3.5HG0524250) and *Xantha-h* (*Xnt-h*, HORVU.MOREX.r3.7HG0738240). They were located inside extensive genomic regions that carried multiple breeding signatures and other genes targeted by domestication and breeding. The *Xantha-g* and *-h* loci encode two out of three subunits of magnesium chelatase, the enzyme that inserts Mg^2+^ into protoporphyrin IX to initiate chlorophyll biosynthesis [98]. The *Xantha-h* gene encodes the 38–42 kDa HvCHLI subunit, while *Xantha-g* encodes the 70–78 kDa HvCHLD subunit [99,100]. Mutations in either of the two genes disrupt Mg-chelatase activity, placing the block at the chelation step and leading to a chlorophyll-deficient phenotype [98]. The genes differ in allelic spectrum. For instance, some *Xantha-g* alleles are leaky: *xan-g45* accumulates protochlorophyllide and forms normal prolamellar bodies, whereas non-leaky alleles lack them [98,101]. At *xan-h*, by contrast, all classical mutants examined were non-leaky, and heterozygotes for these alleles show slight semi-dominance, reducing chlorophyll content by 14–18% [102].

#### 3.2.7. Seed Dormancy

Breeding signatures analysis detected two major barley seed dormancy loci, *Qsd1* (HORVU.MOREX.r3.5HG0481320) and *Qsd2* (HORVU.MOREX.r3.5HG0537440). Both map to the 5H chromosome in different regions: the one containing *Qsd2* carried Tajima’s D for both two-rowed and six-rowed barley, where the two-rowed group showed a recent selective sweep; *Qsd1* was detected by nSL signals, indicating a recent selection process in both groups. *Qsd1* encodes a 494 amino acid alanine aminotransferase expressed specifically in the embryo [103]. *Qsd2* encodes a distinct 523 amino acid protein that, in combination with Qsd1, is required for normal early grain germination [104]. Both genes display long-dormancy (*qsd1*, *qsd2*) and short-dormancy (*Qsd1*, *Qsd2*) alleles, where the reduced-dormancy one almost completely dominates over the long-dormancy allele [103]. The Cas9-mediated premature stop codons in *qsd1* and *qsd2* mutants showed a strongly prolonged grain dormancy [104]. The short-dormancy allele originated from domesticated barley that carried the wild long-dormancy allele and became a desirable trait, particularly in the malting industry, where producers favor more controlled seed germination [103]. Nevertheless, long-dormancy alleles remain prevalent in barley cultivated in areas with warm and rainy weather during grain maturation because it provides more resistance to preharvest sprouting [105,106].

## 4. Materials and Methods

### 4.1. Barley Panel

The studied panel of domesticated spring barley maintained in the GeneAgro seed bank of the Institute of Cytology and Genetics of the Siberian Branch of the Russian Academy of Sciences (https://ckp.icgen.ru/plants/ accessed on 16 October 2026) consisted of 218 accessions in total, where 192 were Russian and 26 were foreign (Supplementary Table S3). The panel included 156 two-rowed and 59 six-rowed accessions and three with an unidentified row type. It also comprised cultivars and breeding lines created in the second half of the twentieth century and up to the present decade and landraces. The cultivation area of the panel accessions covers almost all Russian territories and a wide range of environments.

### 4.2. DNA Extraction and Library Preparation 

Total genomic DNA was isolated from young leaf tissue of two plants per genotype (pooled together during homogenization) using the modified sodium bisulfite protocol described in [107]. DNA quality and concentration were evaluated by agarose gel electrophoresis and quantified on a NanoDrop 2000 Spectrophotometer (Thermo Scientific™, Wilmington, NC, USA). Genotyping by sequencing (GBS) was carried out at the Institute of Cytology and Genetics, the Siberian Branch of the Russian Academy of Sciences using the protocol described in [104]. Briefly, following the protocol of Poland et al. [108], genomic DNA was digested with the restriction enzymes *MspI* and *PstI* (SibEnzyme, Novosibirsk, Russia). GBS libraries were constructed by ligating unique barcoded adapters to the digested fragments, followed by PCR amplification. Libraries were pooled in equimolar ratios, and fragments within the 210–520 bp range were size-selected using a BluePippin system (SageScience, Beverly, MA, USA). Sequencing was performed on an Illumina NextSeq 550 instrument using a 150 bp single-end configuration. 

### 4.3. Genotyping-by-Sequencing (GBS) Data Processing

To perform variant calling, raw reads were submitted to the updated version of the GBS-DP pipeline [109]. Novel features included read quality control and trimming, basic statistics analysis of merged data with plots, and optional LD pruning with threshold customized by user. The first modules analyzed the read quality and then removed adapter sequences with low-quality bases using FastQC v 0.12.1 and fastp v0.24.0 [110]. Next, reads were aligned to the reference genome Morex v2 [111] using BWA MEM [112]. The variant calling was conducted with samtools v1.6 and bcftools v1.9 [113]. Quality thresholds included mapping MAPQ > 20, sequencing depth DP > 3, genotyping quality > 20.

For further work, only biallelic single-nucleotide polymorphisms (SNPs) were retained. Using the plink2 (v2.00a5.12LM) [114], we eliminated SNPs with missing rate > 0.2. After that, individuals with more than 0.2 missing data were also removed. Finally, we saved variants with a minor allele frequency (MAF) > 0.05. The resulting marker density across the barley genome was visualized using the R package “CMplot” [115]. 

### 4.4. Population Structure Analysis 

It was shown that population stratification might artificially inflect the linkage disequilibrium (LD) estimation [116]. To overcome the issue, we used the PCAone tool v0.6.0 [117] that accounts for population stratification during linkage matrix calculations. Thus, it allows maintaining more SNPs. The tool requires a user-defined number of principal components that capture the population stratification. To determine the number of PCs, we first performed principal component analysis (PCA) using the R package “LEA” [118] on unpruned data filtered by missing rate and MAF. Visual examination of scree plot (Supplementary Figure S5) resulted in two PCs describing the most significant part of the population structure. Therefore, to prune data we applied parameter –k 2 (command example: “PCAone -b {plink bfile} -k 2 --ld -o {adjusted} to calculate residuals”) and r^2^ threshold 0.2 to PCAone calculation of LD matrix (command example: “PCAone -B {adjusted}.residuals --match-bim {adjusted}.mbim --ld-r2 0.2 --ld-bp 1000000 -o {LD output prefix}”). 

Next, based on LD-pruned data, we performed PCA and phylogenetic tree construction via neighbor-joining algorithm implemented in TASSEL5 [119]. The tree visualization was performed with R package “ggtree” [120]. To explore ancestry composition of GeneAgro accessions, we applied the STRUCTURE algorithm implemented in the ADMIXTURE v 1.3.0 tool [121]. It estimates individual ancestry proportions and allele frequencies across hypothetical ancestral populations. We ran the ADMIXTURE for different numbers of ancestral clusters K (K = 1–12), repeating the analysis 3 times with different random seeds per K. To find the optimal K, we applied the Cross-Validation (CV) error algorithm implemented in ADMIXTURE. Because ADMIXTURE results describe genetic stratification at increasing levels of resolution rather than identifying a single true number of ancestral populations [122], we evaluated K based on mean CV error, stability across replicates (standard deviation of CV error), and the biological interpretability of the inferred clusters.

### 4.5. Analysis of GeneAgro Diversity in the Context of Worldwide Domesticated Barley

For studying the genetic diversity of domesticated barley samples from the GeneAgro panel within the global context, we used the panel of 19,778 worldwide domestic barley accessions generated by the Leibniz Institute of Plant Genetics and Crop Plant Research (IPK, Gatersleben, Germany) https://commons.datacite.org/doi.org/10.5447/ipk/2020/24 [123]. We combined these data with biallelic variants that underwent the missing rate filtration with threshold 0.2 from GeneAgro panel using the bcftools isec function. The key parameter was -c none, which allowed us to select variants with overlapping positions and corresponding reference and alternative alleles. Prior to PCA, we performed quality control on the merged dataset using plink2. We removed SNPs and samples with more than 0.2 missing data and SNPs with MAF < 0.05. Due to the large size of the dataset, we conducted PCA with plink2 using resources provided by the Center for Collective Use ‘Bioinformatics’ of the Institute of Cytology and Genetics SB RAS and applied a custom Python 3.6.15 script to visualize the results.

### 4.6. Breeding Signatures Analysis

To find genomic signatures of barley breeding in Russia, we performed a genome-wide screening on the GeneAgro collection. Because breeding direction is frequently associated with row type, we divided the whole population into two- and six-rowed barley [124].

The analysis included site frequency spectrum (SFS) calculation and haplotype-based statistics such as Tajima’s D test, fixation index Fst, and estimation of segregating sites by length (nSL) within groups and between groups. Tajima’s D measures the deviation from neutral evolution for the provided sequence by comparing pairwise nucleotide diversity and number of segregating sites [125]. Extreme negative value results from the excess of rare variants and usually indicate a recent selective sweep. Meanwhile, an extreme positive value suggests either balancing selection or a bottleneck event. The Fst measures genetic divergence between two sequences based on allele frequencies [126]. Minimum of 0 means absence of differentiation, and maximum of 1 indicates extremely high divergence level.

Haplotype-based methods are robust in detecting recent and ongoing positive selection that often acts to increase haplotype homozygosity. The nSL quantifies the length of a shared haplotype homozygosity segment between two haplotypes by counting the number of mutations present in the other haplotypes within the same genomic region [127]. Relative to reference, extreme positive and negative values indicate the hard or soft sweep targeting the alternative and reference alleles, respectively. The cross-group metric XP-nSL compares haplotypes between groups and detects genome regions where selective sweep occurred in one group but not the other [128]. Significant positive values suggest breeding pressure on the genome region in the target group, while negative ones indicate selective sweep occurred in the reference group.

SFS metrics may be overestimated on the data filtered by the harsh MAF threshold [129]. Therefore, we used biallelic SNPs of GeneAgro accessions that underwent missingness filtration > 0.2 and MAF filtration with milder threshold 0.01. Next, the filtered GeneAgro data was divided into two- and six-rowed varieties, and then, only Russian ones were selected for the further analysis. To detect loci deviated from neutral evolution, we assessed Tajima’s D separately for each group with VCF-kit v 0.2.6 with window size 500,000 bp and window step 50,000 bp [130]. Using vcftools v 0.1.17 [131], we calculated the Fst index with the same window size and window step thresholds to search for loci differentiating between six- and two-rowed samples.

Prior to homology-based analysis, we conducted the phasing and imputation procedures by BEAGLE 5.5 [132] on unpruned data with effective population size parameter ne = 100 recommended for crops and breeds [133]. The used command example: “beagle gt=file.vcf out={out_prefix} gp=true ne=100 threads=8”.

To detect putative selective sweeps that describe the breeding history of two- and six-rowed barley in Russia, we applied a statistic that assesses the number of segregating sites by length (nSL) using selscan v2.1.1 [134]. The nSL was applied per each row type group, and XP-nSL was estimated between groups (the six-rowed group was set as reference population). On the first step, selscan calculated the nSL/XP-nSL metric for each window with maximum allowed distance between neighbor SNPs --max-gap 1,000,000 bp (command example: “selscan --vcf ${chrom}.vcf.gz --map results_${chrom}.map --skip-low-freq --out results_${chrom} --nsl --max-gap 1,000,000”). Next, raw nSL and XP-nSL scores were normalized using selscan norm (command example: “selscan/src/norm --nsl --files *nsl.out”). The nSL was normalized within 100 alternative-allele-frequency bins, because raw nSL correlates with allele frequency; XP-nSL was normalized genome-wide. Normalized scores, therefore, have mean 0 and standard deviation 1, and the threshold |score| > 2 corresponds to two standard deviations of the empirical distribution rather than an arbitrary cut-off.

### 4.7. Search of Candidate Genes Targeted by Breeding

To detect candidate genes under breeding pressure, we applied a two-step procedure. The first step included extraction of candidate regions obtained by each statistic. In the second step, we performed a gene search inside the significant regions overlapping between two or more selective signals.

For haplotype-based metrics nSL and XP-nSL, we derived SNPs with normalized values more than 2 or less than –2. Then, using the Python library SweepCluster [135], we conducted SNP clustering with parameters -eps 5,000,000 (the neighborhood distance threshold for any two SNPs) and -min_num 3 (minimum number of SNPs in a neighborhood for a SNP to be considered as a core SNP). The resulting regions were considered the most reliable.

We implemented a more strict approach to extract candidate regions from the frequency-based metrics because they may be sensitive to population structure, bottleneck events and allele frequency filtration [129,136]. Firstly, we maintained only robust bins containing more than 10 SNPs. Secondly, we identified bins with top 5% unique extreme Fst values. The same filter was applied to Tajima’s D negative and positive values. Next, using bcftools, we extracted SNPs from significant bins and performed clustering with the same SweepCluster parameters as above. 

These thresholds are empirical and do not constitute formal statistical significance. We therefore refer to the resulting regions as candidate regions. Requiring agreement between frequency-based (Tajima’s D, Fst) and haplotype-based (nSL, XP-nSL) statistics follows the recommendation that combining statistics sensitive to different features of the data improves discrimination in non-equilibrium populations [137]. 

To reveal candidate genes subjected to breeding pressure, we relied on the most well-known functionally characterized genes [22]. In the Jiang et al. work, gene IDs were annotated based on assembly Morex v3. To find their coordinates on assembly Morex v2, we extracted the nucleotide sequences using the Ensembl biomart platform with dataset “MorexV3_pseudomolecules_assembly” https://plants.ensembl.org/biomart/martview/ accessed on 16 October 2026. Then, sequences of these genes were used as queries for BLAST v 2.16.0+ search against the TRITEX Morex v2 assembly with evalue 10^−17^ to decrease noisy alignments. Next, to ensure gene coordinates, mismatch <= 1, evalue < 10^−20^ and length > 500 were applied on the results. Finally, we selected genes whose best alignment coordinates (highest identity percent and bitscore) were inside the loci under selection or in their close proximity (up to 5 Mb).

## 5. Conclusions

In this work, we conducted the first comprehensive study of genetic diversity and breeding signatures of barley from the Russian GeneAgro seed bank as a foundation for the future identification of adaptive alleles. Spike row type was the main factor shaping the genetic composition of the panel, and two-rowed and six-rowed barley showed both shared and row-type-specific selection signatures.

Regions with selection signatures contained candidate genes known to control spike architecture (*HvAP2L-H5/Int-m*, *HvCMF4*, *HvCOM1*, *HvRAW1*, *HvDEP1* and *Trd1*) and plant morphology genes (*HvDIM*, *HvDL/dl*, *ELI-A*, *Lnt1*). Breeding also targeted genes that underlie major yield-associated traits such as flowering time and fertility (*HvPRR37/Ppd-H1*, *HvLEAFY/mov5*, *HvMLH3/des10*, *HvPHYC/eam5*, *Vrn-H1*, *Cly1*, *eam8* and *vrn-H3*). Finally, analysis demonstrated extensive breeding on grain color (*Ant2*, *Ant17*, *Ant28* and *Ant30 genes*), antifungal resistance (*Rpg5*, *Rpg4*, *Rph3*, *Rph1*, *RAR1* and *ROR2*), photosynthesis (*Xantha-g* and *Xantha-h*), and seed dormancy (*Qsd1* and *Qsd2*). We hypothesize that some of these regions reflect the history of Russian barley breeding for adaptation to local growing conditions, pathogen pressure or malting quality. However, the proximity of a selection signal to a known gene does not show which functional alleles are present in the collection, whether they affect the associated traits, or when selection took place. Testing these hypotheses will require allele-level characterization of the candidate genes, phenotypic association studies and analysis of dated historical material.

In the context of one of the major worldwide barley collections, the GeneAgro panel improves the representation of Russian barley, specifically two-rowed and spring types. These findings suggest that Russian germplasm may contribute novel variants to the available genetic resources. 

## Figures and Tables

**Figure 1:**
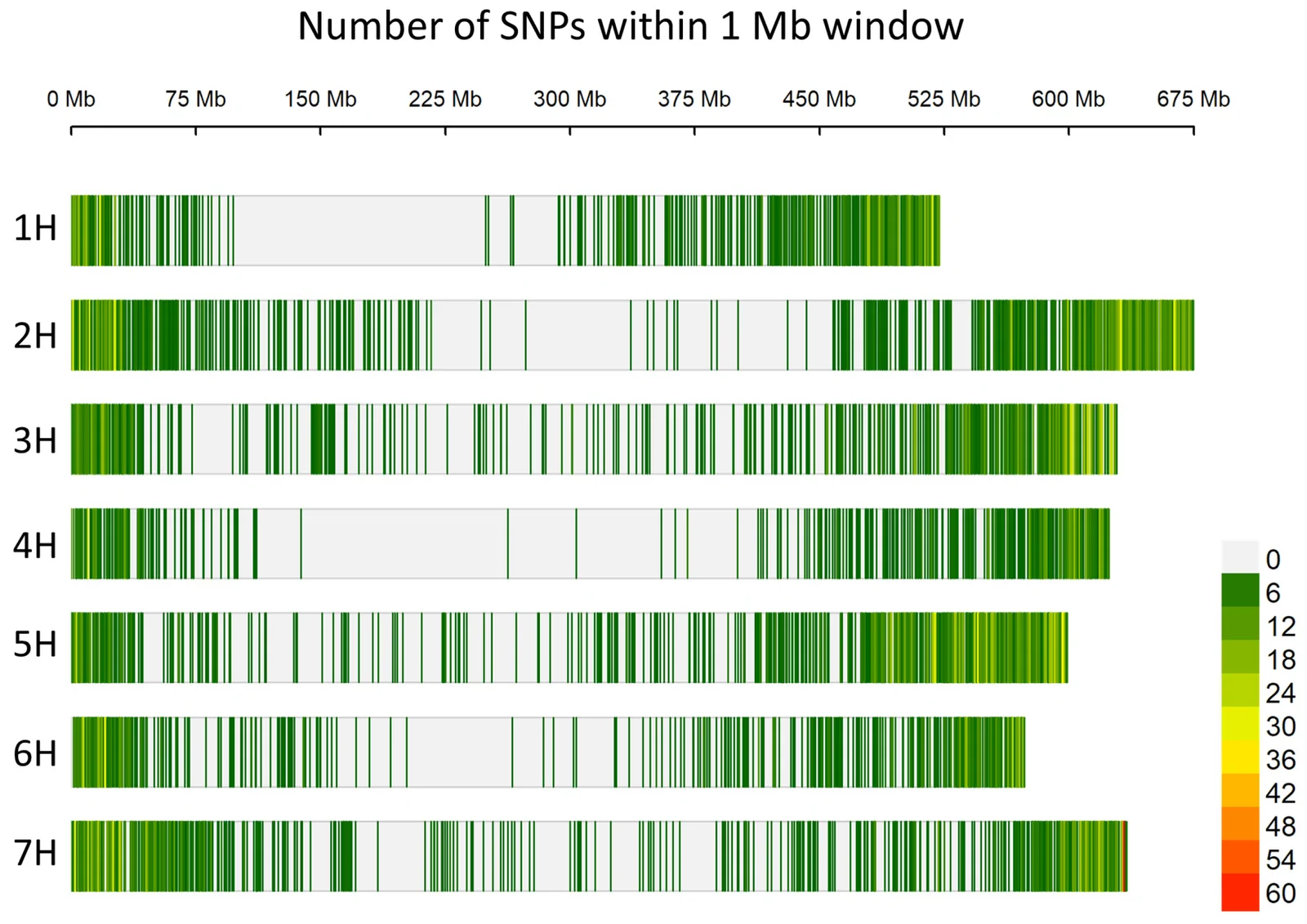
SNP density plot of SNPs within 1 Mb window across barley chromosomes. The dataset includes 9250 SNPs that passed the quality filters. Color bar represents the number of SNPs within a 1 Mb window, going from gray and dark green (zero and low density) to red showing regions carrying the most SNPs.

**Figure 2:**
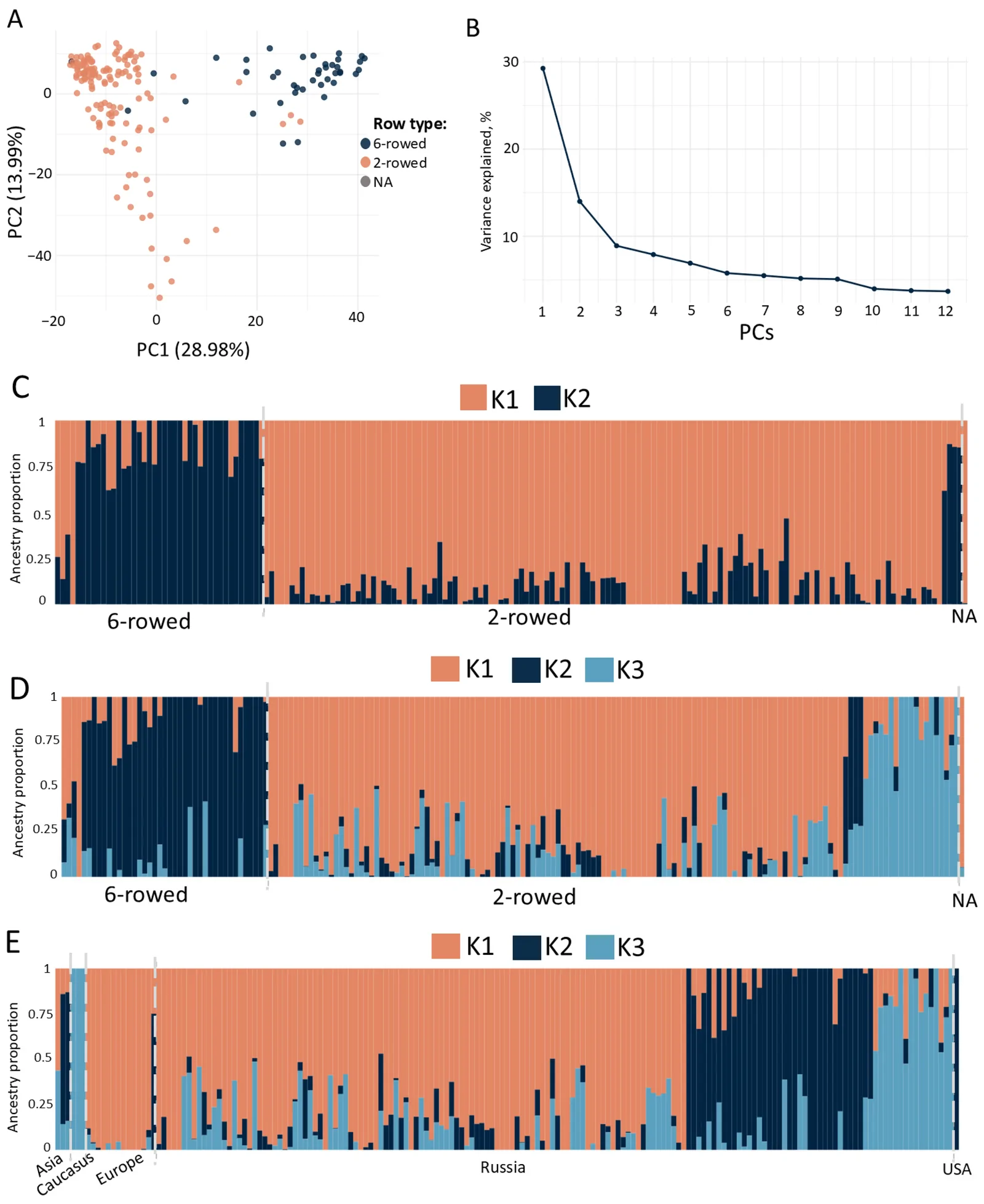
Population structure analysis results of GeneAgro panel based on a filtered and pruned dataset of 179 accessions and 3181 SNPs. (**A**) PCA scatter plot representing the first two principal components. (**B**) PCA scree plot of percentage of variance (y-axis) explained by each principal component settled along the x-axis. (**C**–**E**) Admixture bar plots representing ancestry proportion (y-axis) of accessions (x-axis). (**C**) K = 2, accessions are grouped by row type. (**D**) K = 3, accessions are grouped by row type. (**E**) K = 3, accessions are grouped by geographic region of origin (accession order changed and is shown in Supplementary Figure S6A,B).

**Figure 3:**
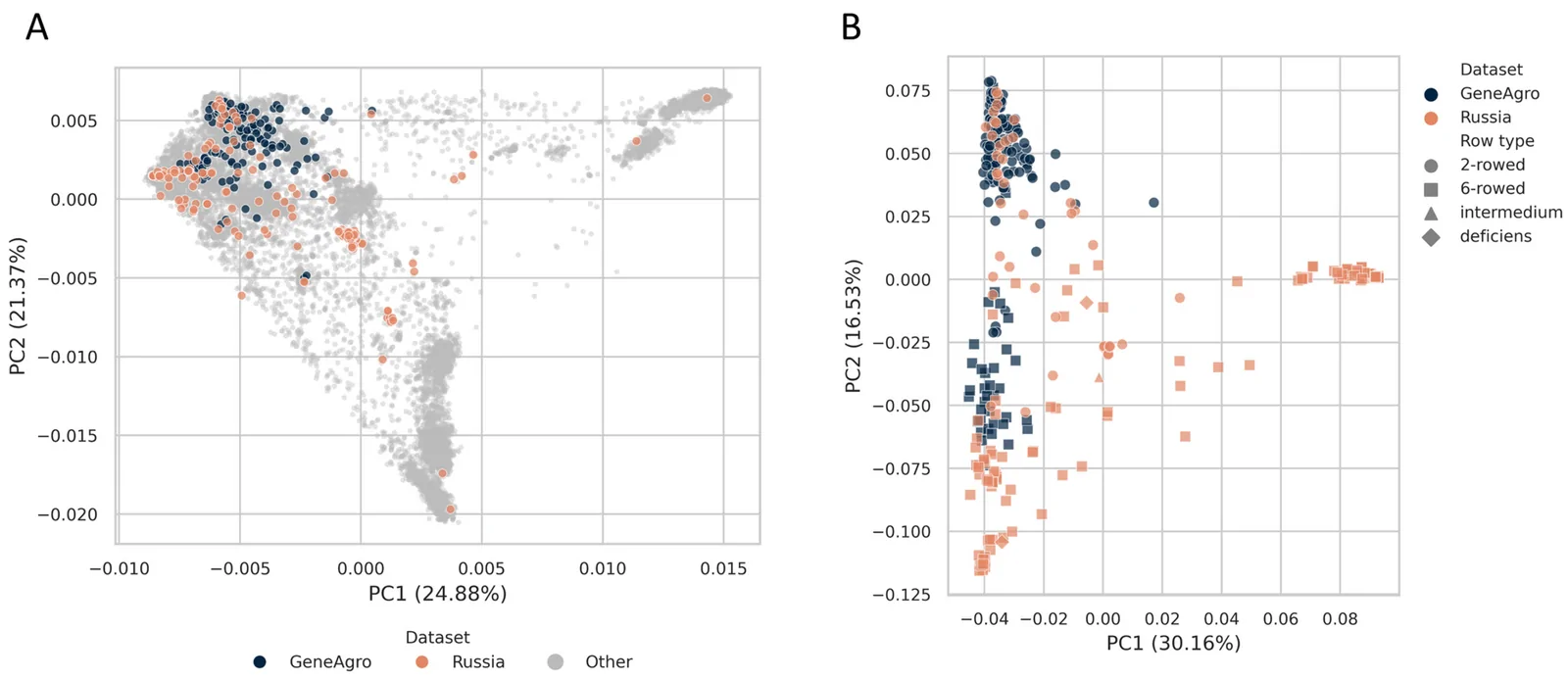
PCA results of GeneAgro and IPK panels combined. (**A**) Scatter plot demonstrates sample clusterization of the whole dataset, where the GeneAgro panel is dark blue and the Russian samples from the IPK panel are orange. Accessions from other databases are gray. (**B**) Scatter plot shows PCA results based on Russian-only accessions from the combined dataset. Orange dots are Russian accessions from the IPK dataset; dark-blue dots represent the GeneAgro panel. Circles—two-rowed barley, squares—six-rowed barley, triangles—deficiens row type, rhombus—intermedium row type.

**Figure 4:**
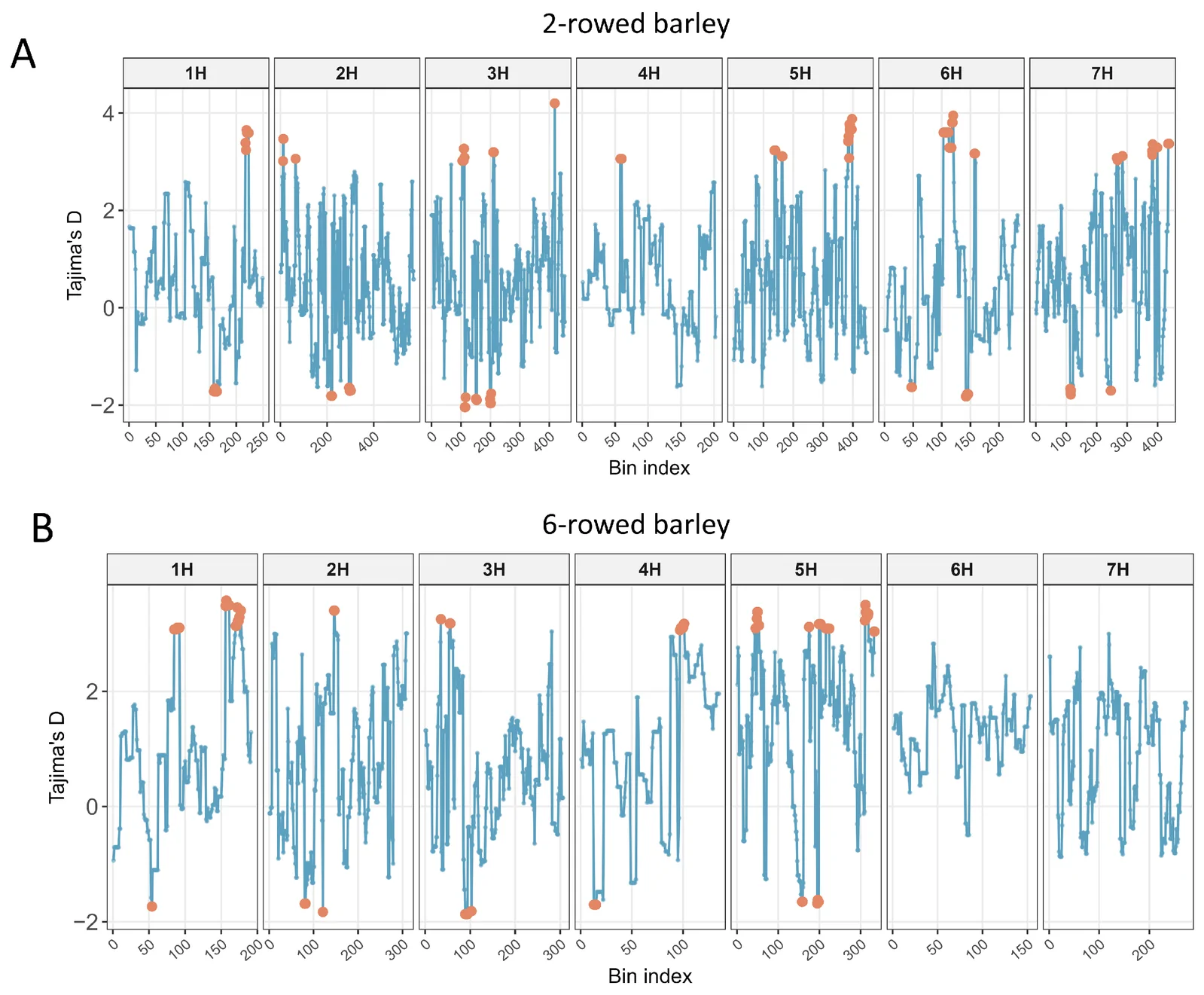
Tajima’s D analysis results for two-rowed (**A**) and six-rowed (**B**) barley. The x-axis represents bins of 500 kb size in ascending order along the chromosome. Orange dots represent candidate regions that rank in the top 5% extreme Tajima’s D values and contain more than 10 SNPs.

**Figure 5:**
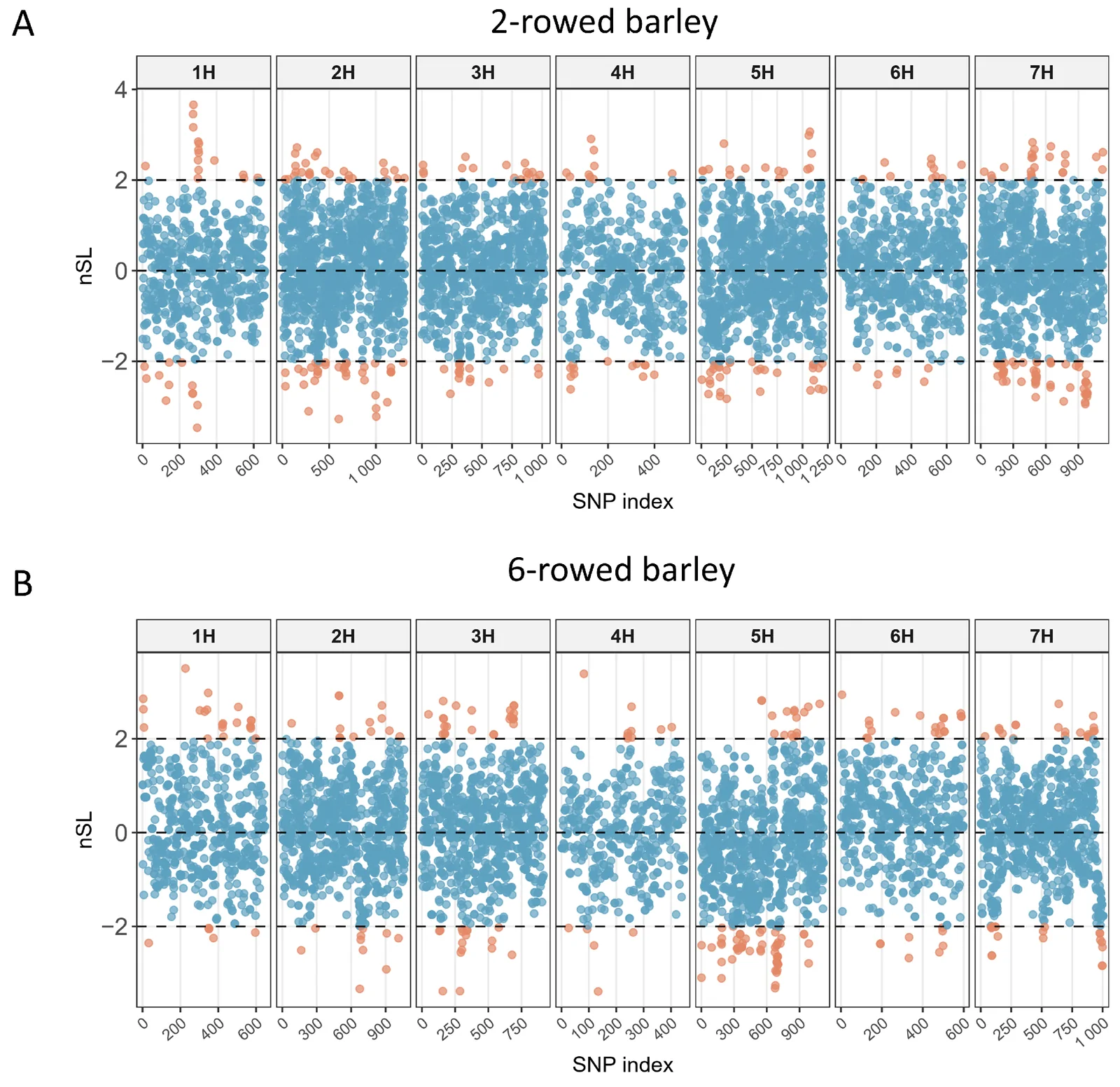
Sweeps for two-rowed (**A**) and six-rowed (**B**) barley obtained by haplotype-based statistic nSL. The x-axis represents the SNP index in ascending order along the chromosome, and the y-axis shows normalized nSL values. Orange dots represent candidate SNPs with |nSL| > 2; blue dots represent all other SNPs (|nSL| ≤ 2). Dashed lines indicate the thresholds of ±2. The in-group haplotype-based statistics effectively detect current selective sweeps when allele under selection is not fixed yet. However, these methods fail to reveal selection events if the allele is fixed in all group members. Therefore, to increase the power of breeding signal analysis, we implemented cross-population approaches capturing differentiation between groups.

**Figure 6:**
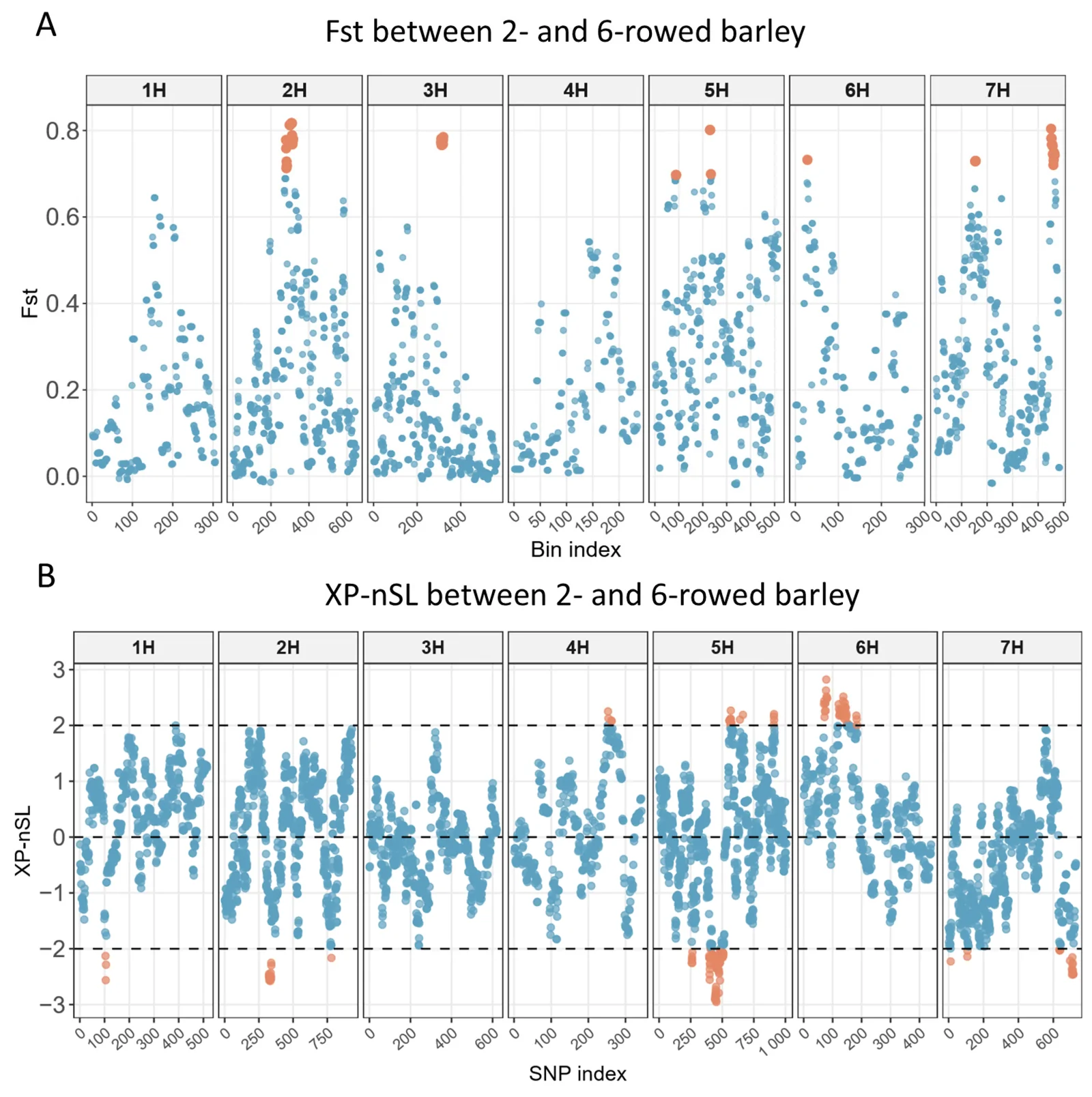
(**A**) The fixation index Fst between two-rowed and six-rowed Russian barley. The y-axis represents the Fst value from 0 to 1; the x-axis represents bins of 500 kb size in ascending order along the chromosome; orange dots are candidate regions containing more than 10 SNPs and rank top 5% among Fst values higher than average. (**B**) The XP-nSL between two-rowed and six-rowed Russian barley. The y-axis represents the XP-nSL statistic; the x-axis represents SNP in ascending order along the chromosome; orange dots are significant regions. The y-axis shows normalized XP-nSL values. Orange dots represent candidate SNPs with |XP-nSL| > 2; blue dots represent all other SNPs (|XP-nSL| ≤ 2). Dashed lines indicate the thresholds of ±2.

**Table 1:** Candidate genes located near genomic regions of breeding signatures analysis presented in Supplementary Table S2.

Gene	Gene ID Morex v3	Trait	Gene Product
*Trd1*	HORVU.MOREX.r3.1HG0091840	Spikelet character	GATA transcription factor
*eam8*	HORVU.MOREX.r3.1HG0095050	Flowering time	Circadian clock regulator, early flowering 3
*Rph1*	HORVU.MOREX.r3.2HG0100980	Leaf rust resistance	NLR protein
*ELI-A*	HORVU.MOREX.r3.2HG0105830	Leaf shape	Uncharacterized protein containing an RNaseH-like domain
*HvPRR37/Ppd-H1*	HORVU.MOREX.r3.2HG0107710	Flowering time	Pseudo-response regulator (PRR)
*HvbHLH1/Ant2*	HORVU.MOREX.r3.2HG0188710	Pigmentation	Basic helix-loop-helix (bHLH) transcription factor
*RAR1*	HORVU.MOREX.r3.2HG0189710	Powdery mildew resistance	CHORD domain zinc binding protein
*HvLEAFY/mov5*	HORVU.MOREX.r3.2HG0194240	Fertility	Transcription factor LEAFY
*Ant17*	HORVU.MOREX.r3.2HG0201360	Pigmentation	Flavanone-3-hydroxylase
*Cly1*	HORVU.MOREX.r3.2HG0204770	Fertility	AP2-domain transcription factor
*Lnt1*	HORVU.MOREX.r3.3HG0302040	Tiller number	BELL-like homeodomain transcription factor JuBel2
*Ant28*	HORVU.MOREX.r3.3HG0316260	Pigmentation	R2R3 MYB transcription factor
*HvCMF4/tst2.b*	HORVU.MOREX.r3.4HG0411680	Grain and spikelets number	CCT motif family protein
*HvCOM1*	HORVU.MOREX.r3.5HG0479720	Spike branching	Grass-specific TCP transcription factor
*HvDEP1*	HORVU.MOREX.r3.5HG0480200	Grain character	Heterotrimeric G-protein γ-subunit
*Qsd1*	HORVU.MOREX.r3.5HG0481320	Seed dormancy	Alanine aminotransferase
*HvRAW1*	HORVU.MOREX.r3.5HG0502720	Awn roughness	Cytokinin riboside 5′-monophosphate phosphoribohydrolase
*HvMLH3/des10*	HORVU.MOREX.r3.5HG0503940	Fertility	MutL-Homolog 3 protein
*HvPHYC/eam5*	HORVU.MOREX.r3.5HG0511090	Flowering time	Phytochrome C
*Vrn-H1*	HORVU.MOREX.r3.5HG0511210	Flowering time	Apetala-1-like protein
*ROR2*	HORVU.MOREX.r3.5HG0517040	Powdery mildew resistance	Synaptosome-associated protein-25 receptor, SNARE
*Xantha-g*	HORVU.MOREX.r3.5HG0524250	Photosynthesis	Magnesium chelatase middle-size subunit
*HvAP2L-H5/Int-m*	HORVU.MOREX.r3.5HG0525620	Spike row type	APETALA2-like transcription factor 5
*Ant30*	HORVU.MOREX.r3.5HG0525800	Pigmentation	Chalcone isomerase
*Rpg5*	HORVU.MOREX.r3.5HG0526140	Stem rust resistance	Actin depolymerizing factor-like protein
*Rpg4*	HORVU.MOREX.r3.5HG0526170	Stem rust resistance	NLR protein
*Qsd2*	HORVU.MOREX.r3.5HG0537440	Seed dormancy	Mitogen-activated Protein Kinase Kinase 3 (MKK3)
*Vrn-H3*	HORVU.MOREX.r3.7HG0653910	Flowering time	FLOWERING LOCUS T-like protein
*HvDL/dl*	HORVU.MOREX.r3.7HG0736060	Leaf shape	Arabidopsis thaliana LSH1 and Oryza G1 (ALOG) family protein
*Xantha-h*	HORVU.MOREX.r3.7HG0738240	Photosynthesis	Magnesium chelatase small subunit
*Rph3*	HORVU.MOREX.r3.7HG0741050	Leaf rust resistance	Transmembrane protein
*HvDIM*	HORVU.MOREX.r3.7HG0749940	Culm length	DIMINUTO protein

## Data Availability

The original contributions presented in this study are included in the article/Supplementary Material. Further inquiries can be directed to the corresponding authors.

## Supplementary Materials

The following supporting information can be downloaded at: https://www.mdpi.com/article/10.3390/ijms27209254/s1.

## Abbreviations

The following abbreviations are used in this manuscript:

DNA	Multidisciplinary Digital Publishing Institute
GBS	Genotyping by sequencing
IPK	Leibniz Institute of Plant Genetics and Crop Plant Research
LD	Linkage disequilibrium
MAF	Minor allele frequency
nSL	Number of segregating sites by length
PCA	Principal component analysis
SFS	Site frequency specter
SNP	Single-nucleotide polymorphism
