# Supplementary material for: Application of Genotyping-by-Sequencing for Analysis of Breeding Signatures in Russian Spring Barley Collection
Source: Int J Mol Sci. 2026 Oct 18;27(20):9254. doi: 10.3390/ijms27209254 (PMC13705539; doi:10.3390/ijms27209254)
Supplement: Supplementary file 1 [file ijms-27-09254-s001.zip › Supplementary_figures.pdf]

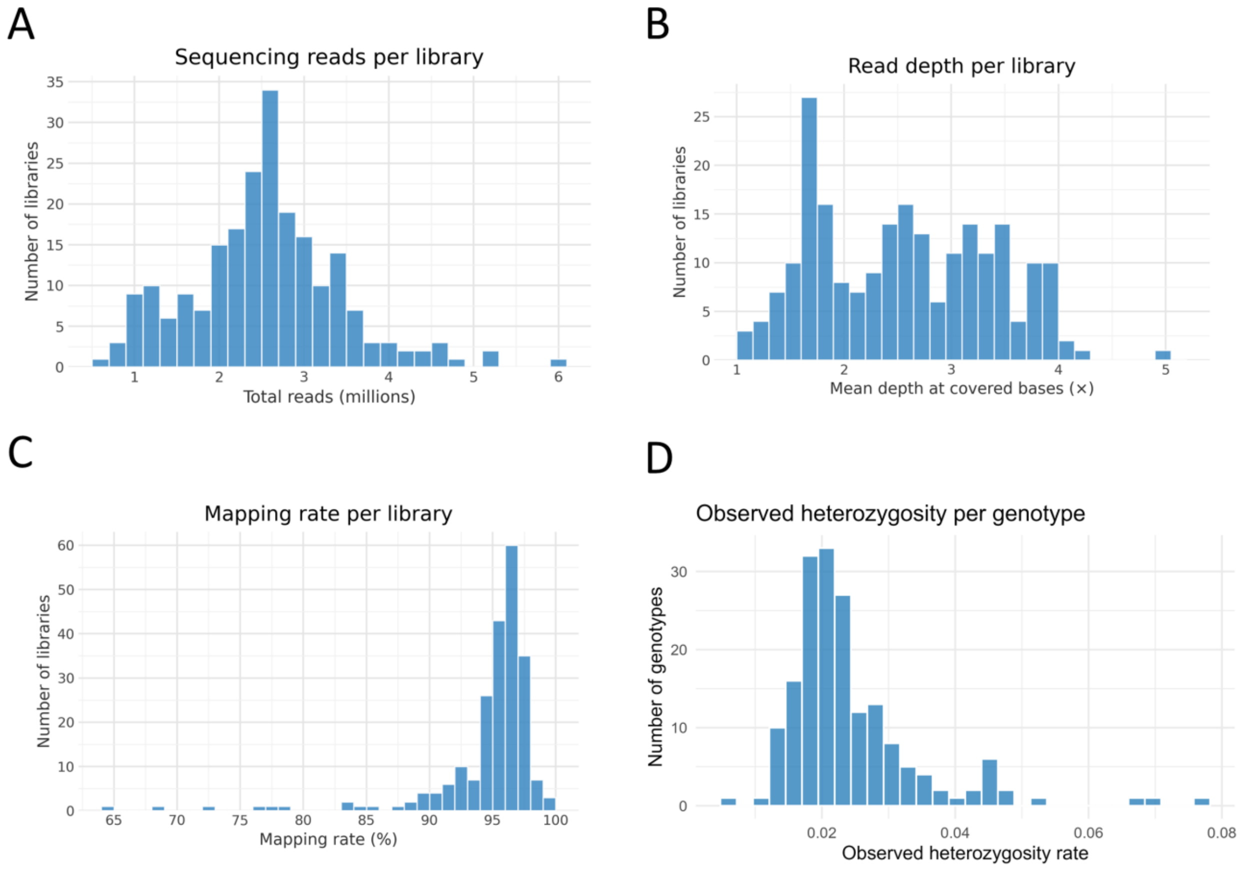

Supplementary Figure S1. Sequencing and genotyping quality statistics. (A) Distribution of total sequencing reads per library. (B) Mean read depth per library, calculated over genomic positions covered by at least one read (MAPQ $\geqslant$ 20), i.e. over the GBS loci rather than the whole genome. (C) Distribution of mapping rates per library, defined as the percentage of reads mapped to the Morex V2 reference genome. (D) Observed heterozygosity per genotype, calculated as the proportion of heterozygous calls among non-missing genotype calls at 18687 filtered SNPs. Panels A–C show all 218 sequenced libraries; panel D shows the 179 genotypes retained for analysis after filtering genotypes with missing data more than 0.2.

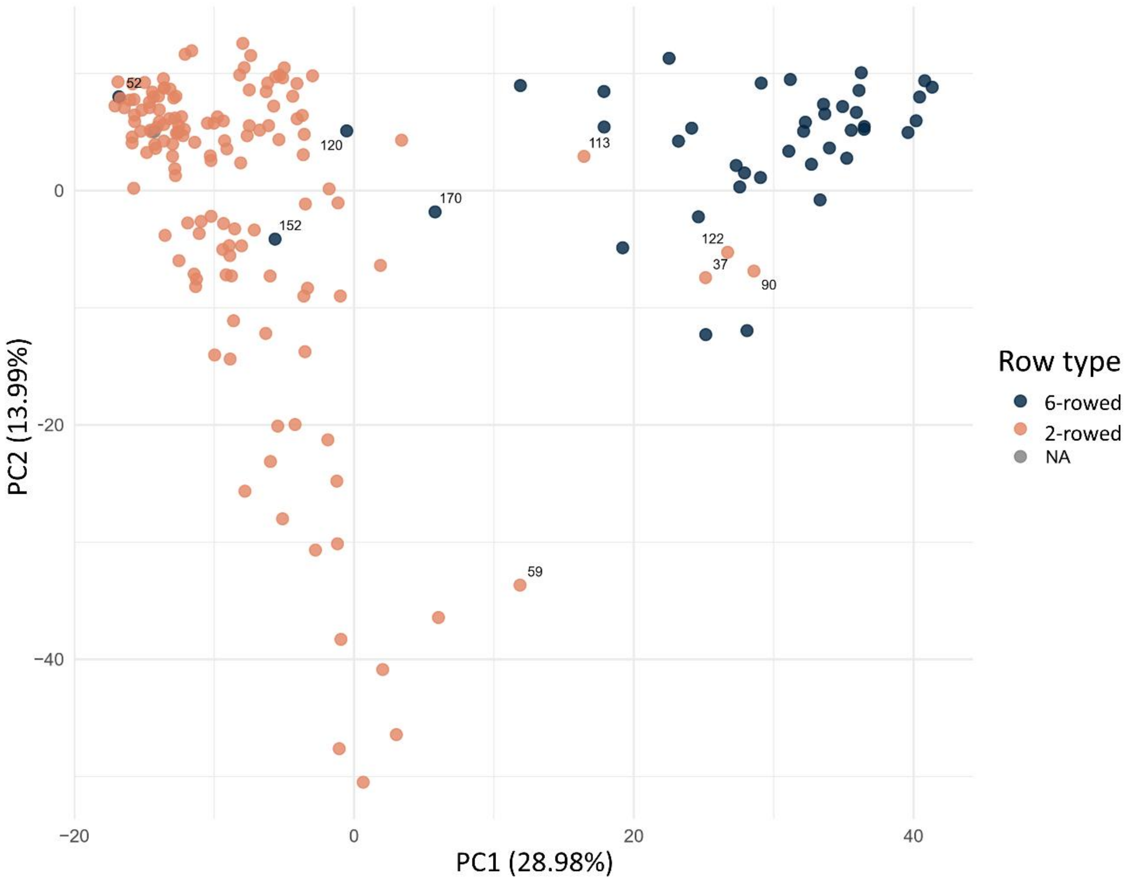

Supplementary Figure S2. PCA results of 179 GeneAgro accessions colored based on row type. Cross-row-type accessions are labeled.

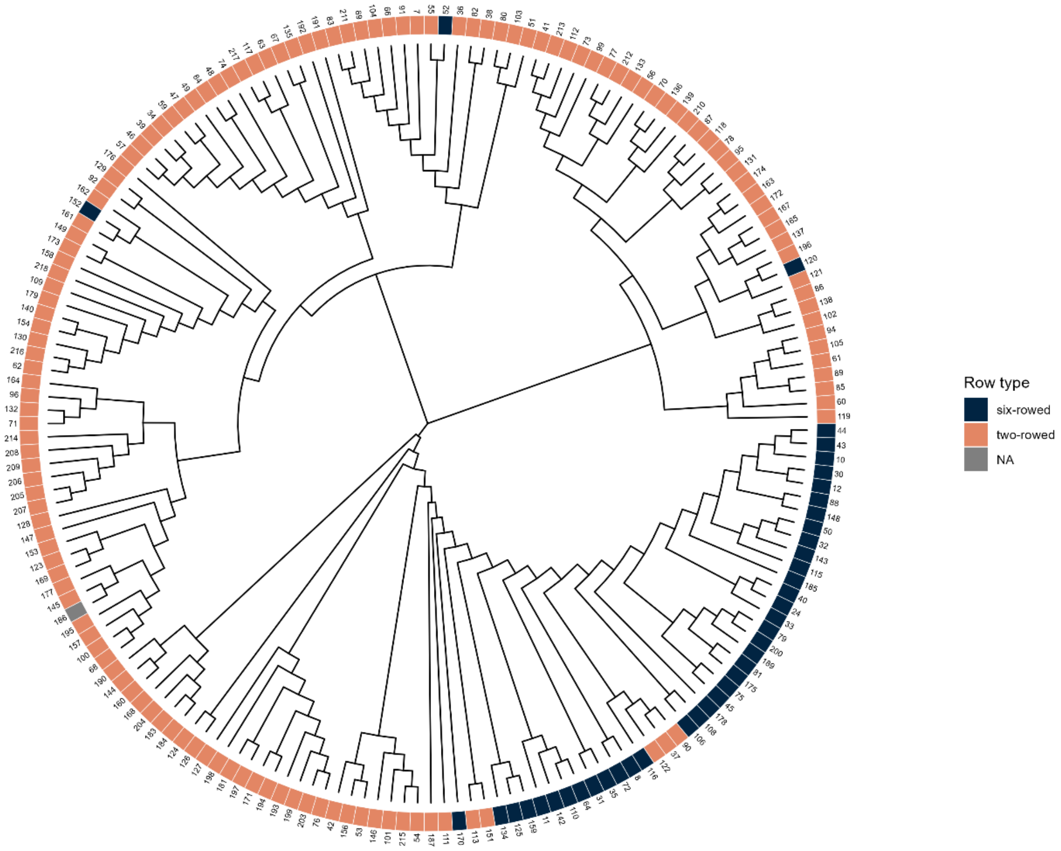

Supplementary Figure S3. Neighbor-joining phylogenetic tree of 179 GeneAgro accessions. Bars are colored according to row type. Orange – two-rowed barley, blue – six-rowed barley.

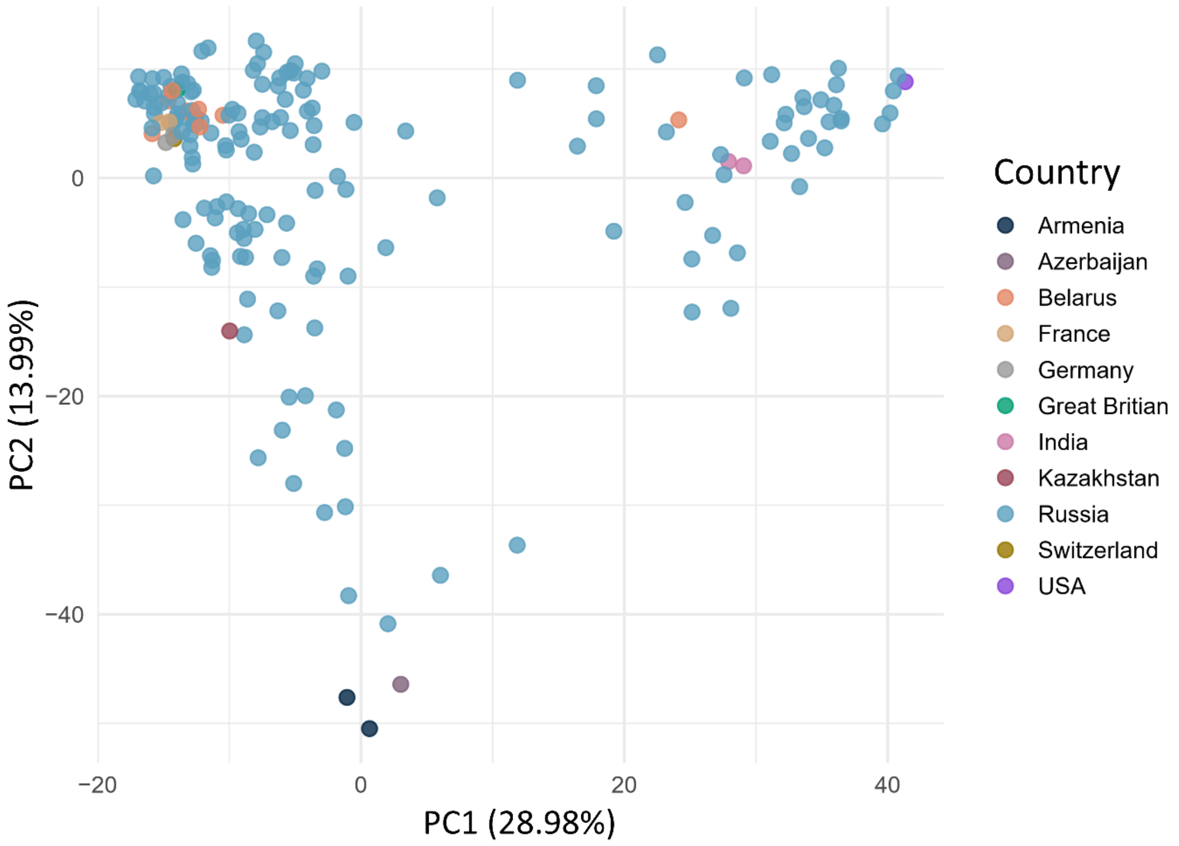

Supplementary Figure S4. PCA results of 179 GeneAgro accessions colored based on the country they were derived from.

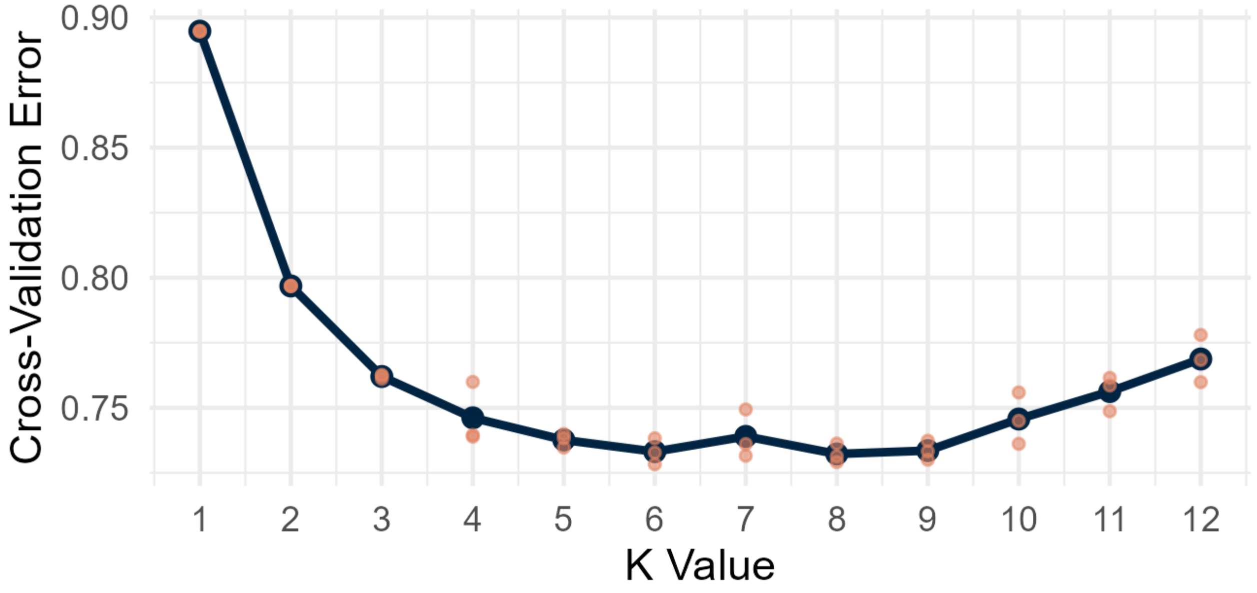

Supplementary Figure S5. Admixture Cross-Validation error calculated for each run (orange dots). The mean error is represented by dark-blue dots.

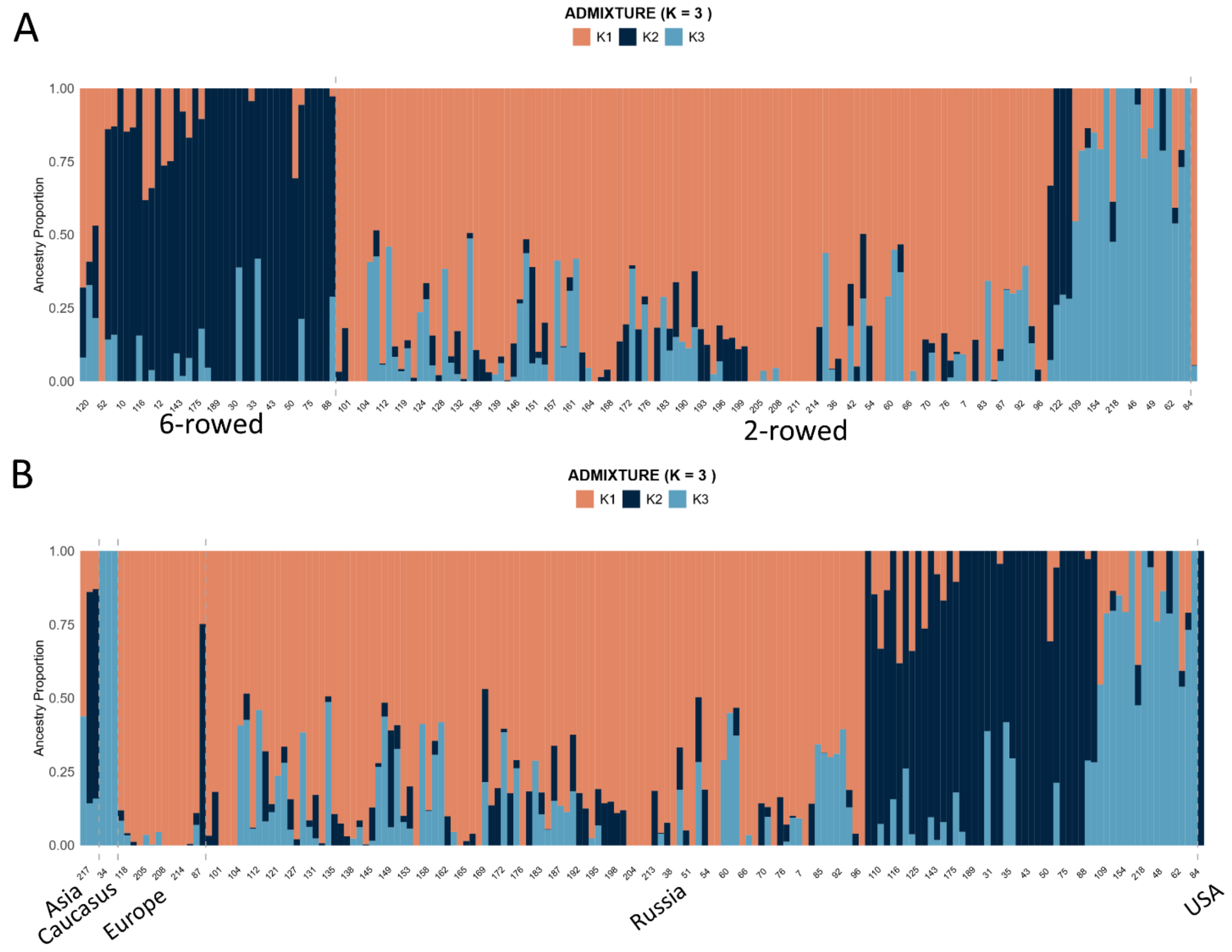

Supplementary Figure S6. Admixture barplots of GeneAgro panel based on filtered and pruned dataset of 179 accessions and 3181 SNPs representing ancestry proportion (Y axis) of accessions (X axis). Every third accession is labeled to demonstrate the order inside clusters. (A) K=3, accessions are grouped by row type and every third accession is shown. (B) K=3, accessions are grouped by geographic region of origin (accession order changed, every third label is shown).

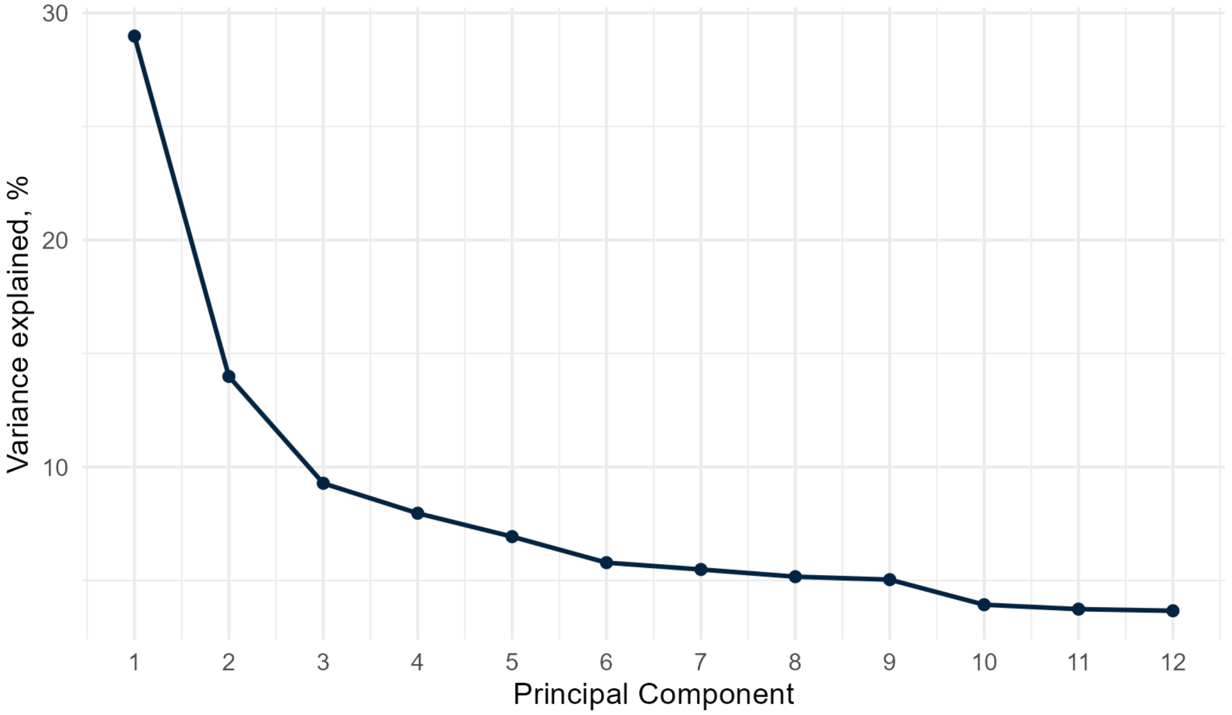

Supplementary Figure S7. Variance explained by principal components of GeneAgro dataset filtered by missing rate and MAF (179 accessions and 9250 SNPs).
